# MiR-SNPs as Markers of Toxicity and Clinical Outcome in Hodgkin Lymphoma Patients

**DOI:** 10.1371/journal.pone.0064716

**Published:** 2013-05-21

**Authors:** Alfons Navarro, Carmen Muñoz, Anna Gaya, Marina Díaz-Beyá, Bernat Gel, Rut Tejero, Tania Díaz, Antonio Martinez, Mariano Monzó

**Affiliations:** 1 Molecular Oncology and Embryology Laboratory, Human Anatomy Unit, School of Medicine, University of Barcelona, IDIBAPS, Barcelona, Spain; 2 Hematology Department, Hospital Clínic, IDIBAPS, Barcelona, Spain; 3 Josep Carreras Leukaemia Research Institute, Barcelona, Spain; 4 Hereditary Cancer Program, Institute of Predictive and Personalized Medicine of Cancer (IMPPC), Badalona, Spain; 5 Department of Pathology, Hospital Clínic, Barcelona, Spain; University College London, United Kingdom

## Abstract

**Background:**

In recent years, microRNA (miRNA) pathways have emerged as a crucial system for the regulation of tumorogenesis. miR-SNPs are a novel class of single nucleotide polymorphisms that can affect miRNA pathways.

**Design and Methods:**

We analyzed eight miR-SNPs by allelic discrimination in 141 patients with Hodgkin lymphoma and correlated the results with treatment-related toxicity, response, disease-free survival (DFS) and overall survival (OS).

**Results:**

The KRT81 (rs3660) GG genotype was associated with an increased risk of neurological toxicity (*P* = 0.016), while patients with XPO5 (rs11077) AA or CC genotypes had a higher rate of bleomycin-associated pulmonary toxicity (*P* = 0.048). Both miR-SNPs emerged as independent factors in the multivariate analysis. The XPO5 AA and CC genotypes were also associated with a lower response rate (*P* = 0.036). XPO5 (*P* = 0.039) and TRBP (rs784567) (*P* = 0.022) genotypes emerged as prognostic markers for DFS, and XPO5 was also associated with OS (*P* = 0.033). In the multivariate analysis, only XPO5 emerged as an independent prognostic factor for DFS (HR: 2.622; 95%CI 1.039–6.620; *P* = 0.041). Given the influence of XPO5 and TRBP as individual markers, we then investigated the combined effect of these miR-SNPs. Patients with both the XPO5 AA/CC and TRBP TT/TC genotypes had the shortest DFS (*P* = 0.008) and OS (*P* = 0.008).

**Conclusion:**

miR-SNPs can add useful prognostic information on treatment-related toxicity and clinical outcome in Hodgkin lymphoma and can be used to identify patients likely to be chemoresistant or to relapse.

## Introduction

Hodgkin lymphoma (HL) is a highly curable B-cell neoplasm characterized by the presence of a relatively small population of malignant tumor cells, known as Hodgkin/Reed-Sternberg (HRS) cells, in a non-neoplastic microenvironment [1]. As the neoplastic HRS cells typically represent <1% of the total infiltrate, crosstalk between the non-neoplastic inflammatory cell infiltrate and the HRS cells is an integral and important aspect of HL[2]. The introduction of MOPP (mechlorethamine, vincristine, procarbazine, and prednisone) was a seminal event in the treatment of HL, attaining complete response rates approaching 84% and long-term disease-free survival (DFS) rates of approximately 66% [3]. Later, however, ABVD (doxorubicin/bleomycin/vinblastine/dacarbazine) was shown to have a superior risk-to-benefit ratio, and ABVD or MOPPABVD (MOPP alternating with ABVD) is now the standard chemotherapy regimen for HL [4]. The treatment of patients with HL is primarily guided by the clinical stage of disease. Stage I–II patients are treated with chemotherapy, mainly ABVD, followed by involved field radiotherapy [5]. In stages III–IV, combination chemotherapy followed by consolidation radiotherapy in selected patients is the standard treatment, with three regimens: ABVD, escalated BEACOPP (bleomycin, etoposide, doxorubicin, cyclophosphamide, vincristine, procarbazine, and prednisone) and STANFORD V (doxorubicin, vinblastine, mechlorethamine, vincristine, bleomycin, etoposide, and prednisone) [6]. While the majority of patients will be cured, treatment-related toxicities are often a cause of late mortality. All three regimens are associated with both acute and long-term toxicities, including neutropenia, nausea/vomiting, sensory neuropathy, bleomycin-associated pulmonary toxicity, and cardiomyopathy [7]. Moreover, a non-negligible percentage (20%) of patients will relapse or will be refractory after first-line treatment[8]. Biomarkers to accurately identify patients with a high risk of treatment failure or recurrence would thus be a useful tool in the management of HL.

In recent years, the microRNA (miRNA) pathway has emerged as a crucial system for the regulation of tumorogenesis, where miRNAs can act as either tumor suppressor or oncogenes [9]. These small RNA molecules regulate numerous processes in the tumor cell mainly by inhibiting the translation of multiple messenger RNAs [10]. In HL, miRNA expression has been assessed in lymph nodes [11], in microdissected HRS cells [12], and in HL cell lines [13], [14]. One of several deregulated miRNAs, miR-135a, was shown to have prognostic significance [15]. miRNA expression is known to be deregulated in tumors due to various mechanisms, such as chromosome alterations, mutations, deletions, methylation or polymorphisms [16].

Single nucleotide polymorphisms (SNPs) are DNA variations that occur when a single nucleotide in the genome sequence is altered. Millions of SNPs have been catalogued in the human genome, and their pattern in each individual can help explain the development of disease and response to pathogens, chemicals, drugs and vaccines [17], making them key enablers for personalized medicine [18]. SNPs related to the miRNA pathway, known as miR-SNPs [19], can affect miRNA functions in different ways depending on their location [20] – either by directly impacting miRNA expression levels or by influencing the miRNA-target interaction. SNPs in the promoter sequence of the miRNA gene can alter its transcription, those in the pri- or pre-miRNA sequence can influence miRNA maturation [21], and those regulating the expression of proteins involved in miRNA biogenesis can alter the miRNAome in the cell. SNPs in the mature sequence of the miRNA or in the miRNA target sites are both frequent and specific and can disrupt or alter the miRNA-mediated repression of a target gene [22]. The study of miR-SNPs thus opens a new area of research in cancer biology and clinical oncology, especially as related to risk stratification, response to therapy, and treatment-related toxicity.

SNPs in interleukin genes, DNA repair genes and metabolic enzymes have been associated with risk [23], clinical outcome [24], [25] and treatment-related toxicities [26] in HL. miR-SNPs have been related to clinical outcome in several solid tumors [27], [28] and in myeloma[29]. However, to the best of our knowledge, the role of miR-SNPs in HL has not been investigated. In the present work, we have assessed the presence of eight miR-SNPs in HL patients and explored their potential as markers of treatment-related toxicity and prognosis.

## Results

### Patients

Table 1 shows the main demographic and clinical characteristics for all 141 patients. Median age was 32 years (range, 13–89) and 51.1% were males. The majority (58.9%) had nodular sclerosis. Epstein-Barr Virus was present in 38.1% of the samples. First-line therapy consisted of ABVD in 52% of patients and MOPPABVD in 38%. Median follow-up was 50 months (range, 1–143).

**Table 1:** Patient characteristics and *P*-values for DFS and OS in the univariate analyses.

Characteristic	Value	*N* = 141*N* (%)	*5 year DFS(%)* *(DFS time range)*	*DFS* *P*-value	*5 year OS(%)* *(OS time range)*	*OS* *P*-value
**Sex**	Male	72 (51.1)	71.1 (1 to 135)	0.817	88.9 (2 to 142.4)	0.722
	Female	69 (48.9)	79.3 (3 to 126)		87.4 (2 to 144)	
**Median age (range)**		32 (13–89)				
	<45	110 (78)	77.9 (1 to 135)	0.100	95.7 (9 to 143)	<0.001
	≥45	31 (22)	64.7 (3 to 91)		61 (2 to119)	
**Histology**	Nodular sclerosis	83 (58.9)	77.2 (1 to 135)	0.478	95.1 (9 to 144)	0.001
	Other	58 (41.1)	72.4 (1 to 106)		78.5 (2 to 120)	
**B symptoms**	Yes	59 (41.8)	69.4 (1 to 104)	0.470	75.6 (2 to 127)	<0.001
	No	82 (58.2)	78.6 (3 to 135)		92.7 (11 to 144)	
**Bulky mass**	Yes	29 (20.6)	84.4 (4 to 111)	0.239	90.4 (11 to	0.298
	No	112 (79.4)	72.8 (1 to 135)		127)86.3 (2 to 144)	
**Anemia, Hb levels less than 10^5^ g/L**	Yes	31 (22)	60 (1 to 77)	0.091	70.4 (2 to 127)	<0.001
	No	110 (78)	79.2 (1 to 135)		93.4 (9 to 144)	
**Leukocytosis, more than 15×10^9^/L**	Yes	17 (12.1)	75 (2 to 82)	0.987	88.2 (10 to 92)	0.287
	No	124 (87.9)	75.9 (1 to 135)		88.4 (2 to 144)	
**Lymphocytopenia, <0.6×10^9^/L or <8% of WBC**	Yes	17 (12.1)	57.1 (1 to 78)	0.642	68.8 (2 to 119)	0.003
	No	124 (87.9)	77.6 (1 to 135)		90.7 (2 to 144)	
**Hypoalbuminemia, <40 g/L**	Yes	46 (32.6)	77.6 (1 to 126)	0.656	70.7 (2 to 142)	<0.001
	No	86 (60.9)	73.2 (1 to 135)		94.3 (9 to 144)	
**High LDH level, >450 UI/L**	Yes	40 (28.4)	64.6 (1 to 106)	0.321	78.6 (2 to 119)	0.021
	No	99 (70.2)	78.6 (1 to 135)		92.1 (2 to 144)	
**High B-2-microglobulin level,>25 mg/L**	Yes	27 (19.1)	73.1 (1 to 104)	0.974	68.2 (2 to 112)	<0.001
	No	84 (60)	72.6 (1 to 135)		94.9 (9 to 144)	
**Stage**	early (I–II)	86 (61)	78.5 (1 to 126)	0.148	96.7 (3 to 142)	<0.001
	advanced (III–IV)	54 (38.6)	68 (1 to 134)		74.2 (2 to 144)	
**EBV**	Positive	40 (38.1)	50.8 (1 to 90)	0.480	75 (2 to 106)	0.120
	Negative	65 (46.1)	69.9 (1 to 90)		87.9 (2 to 100)	
	Unknown	36 (15.8)				
**Treatment**	ABVD	73 (52.1)	60.6 (1 to 109)	0.265	95.7 (2 to 116)	0.221
	MOPPABV	53 (37.9)	77.9 (1 to 135)		86.1 (2 to 145)	
	MOPP	8 (5.7)	100 (26 to 120)		85.7 (3 to 142)	
	Other	7 (4.3)			25 (2 to 19)	
**Toxicities**	Neutropenia	47 (33.3)	77.9 (3 to 104)	0.473	79.6 (1–112)	0.018
	Anemia	6 (4.3)	66.7(3 to 91)	0.375	33.3 (2 to 99)	<0.001
	Thrombocytopenia	6 (4.3)	66.7 (3 to 91)	0.658	44.4 (2 to 99)	0.001
	Pulmonary toxicity	7 (5)	68.6 (3 to 88)	0.464	85.7 (16 to106)	0.889
	Neurological toxicity	23 (16.3)	71 (6 to 113)	0.729	95.2 (19 to135)	0.169
	Infectious toxicity	51 (36.2)	80 (3 to 113)	0.375	80 (1 to 123)	0.019

### miR-SNPs, treatment related-toxicity and response

Table 2 shows the genotypic frequencies for all eight miR-SNPs analyzed, both in the present study and as reported in the NCBI SNP database (dbSNP) for the European population.

**Table 2:** Genotypic frequencies in the present study and for the European Population (HapMap-CEU) in NCBI dbSNP.

Gene	Genotype	European population (%)	Present study *N* * (%)	HWE X^2^ (p-value)
***MIR196A2*** ** rs11614913**	CC	33.6	61 (43.6)	0.04 (p = 0.838)
*N* = 140	CT	44.2	62 (44.3)	
	TT	22.2	17 (12.1)	
***MIR149*** ** rs2292832**	CC	55	61 (58.7)	11.37 (p<0.001)
*N* = 104	CT	36	28 (26.9)	
	TT	9	15 (14.4)	
***MIR423*** ** rs6505162**	AA	31	36 (32.7)	2.15 (p = 0.143)
*N* = 110	AC	57.5	47 (42.7)	
	CC	11.5	27 (24.5)	
***MIR146A*** ** rs2910164**	GG	59.3	73 (51.8)	0.41 (p = 0.521)
*N* = 141	CG	34.5	59 (41.8)	
	CC	6.2	9 (6.4)	
***KRT81*** ** rs3660**	CC	36.7	32 (23.05)	0.75 (p = 0.387)
*N* = 139	CG	45	64 (46.05)	
	GG	18.3	43 (30.9)	
***FAM179B*** ** rs1053667**	TT	92.9	95 (92.2)	3.59 (p = 0.058)
*N* = 103	CT	6.2	7 (6.8)	
	CC	0.9	1 (1)	
***XPO5*** ** rs11077**	AA	33.6	25 (19.7)	0.48 (p = 0.487)
*N* = 127	AC	46	67 (52.8)	
	CC	20.4	35 (27.6)	
***TRBP*** ** rs784567**	CC	23.9	40 (28.8)	0.04 (p = 0.837)
*N* = 139	CT	54	68 (48.2)	
	TT	22.1	31 (22.3)	

* In some cases the genotype could not be determined for technical reasons; “*N*” indicates the number of patients genotyped in each case.

HWE, Hardy Weinberg equilibrium.

Among all 141 patients, 33.3% had neutropenia, 4.3% anemia, 4.3% thrombocytopenia, 5% bleomycin-associated pulmonary toxicity, 16.3% neurological toxicity and 36.2% infectious-related toxicity (Table 1). The results of the univariate analysis for the association between treatment-related toxicities and clinical characteristics are shown in Table S1. Patients harboring the KRT81 GG genotype had a higher rate of neurological toxicity than those with the CC or CG genotype (31% vs. 12%; *P* = 0.016). Patients carrying the XPO5 AA or CC genotype had a higher incidence of bleomycin-associated pulmonary toxicity than those with the AC genotype (10% vs. 1%; *P* = 0.048).

The overall response rate was 89.4%, with 119 patients (84.4%) who achieved complete response, 7 (5%) who showed a partial response, and 14 (9.9%) non-responders. The overall response rate dropped to 83% in patients harboring the XPO5 AA or CC genotype but rose to 95.7% in those with the CC genotype (*P* = 0.036).

In the multivariate analysis for neurological toxicity including KRT81 genotype, treatment strategy (ABVD or MOPABV), number of cycles of treatment (≤4 cycles or>4 cycles) and all clinical variables with *P*<0.2 in the univariate analysis (Table S1), KRT81 GG genotype emerged as an independent risk factor (HR, 6.652; 95%CI, 1.330–33.262; *P* = 0.021), together with, ABVD treatment strategy (HR, 0.056; 95%CI, 0.012–0.258; *P*<0.001) and reduced number of cycles (HR, 0.045; 95%CI, 0.003–0.705; *P* = 0.027) (Table 3). In the multivariate analysis for bleomycin-associated pulmonary toxicity, including XPO5 genotype, treatment strategy (ABVD or MOPABV), number of cycles of treatment (≤4 cycles or>4 cycles) and all clinical variables with *P*<0.2 in the univariate analysis (Table S1), XPO5 AC genotype emerged as an independent protective factor (HR, 0.49; 95%CI, 0.006–0.376; *P* = 0.004), together with ABVD treatment strategy (HR, 0.197; 95%CI, 0.059–0.651; *P* = 0.008) (Table 3).

**Table 3:** Multivariate analyses for neurological toxicity and for bleomycin-associated pulmonary toxicity.

		*P*	Hazard Ratio	95% CI
Neurological Toxicity				
	**ABVD treatment**	**p<0.001**	**0.056**	**0.012–0.258**
	**≤4 cycles of treatment**	**0.027**	**0.045**	**0.003–0.705**
	**Female**	**0.002**	**0.057**	**0.009–0.354**
	High LDH	0.625	1.458	0.322–6.602
	EBV	0.322	1.913	0.530–6.902
	**KRT81 GG**	**0.021**	**6.652**	**1.330–33.262**
**Pulmonary toxicity**				
	**ABVD treatment**	**0.008**	**0.197**	**0.059–0.651**
	≤4 cycles of treatment	0.998	0.000	0.00-.
	Anemia	0.878	0.868	0.143–5.268
	High B-2-microglobulin	0.660	1.446	0.280–7.460
	**XPO5 AC**	**0.004**	**0.49**	**0.006–0.376**

### miR-SNPs, DFS and OS

**Figure 1:**
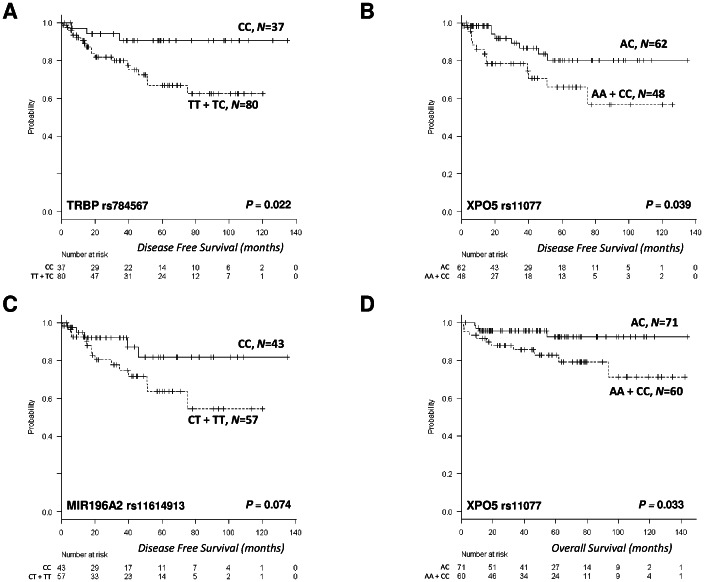
Clinical outcomes according to miR-SNPs. DFS according to TRBP, XPO5 and MIR196A2, and OS according to XPO5. (A) Mean DFS for 37 patients (31.6%) with the TRBP CC genotype was 124 months (95% CI, 112–136) vs. 86.8 months (95% CI, 74–89) for those with the TT or TC genotype (*P* = 0.022). (B) Mean DFS for 62 patients (56.3%) with the XPO5 AC genotype was 114.2 months (95% CI, 101–127) vs. 85.8 months (95% CI, 68–104) for patients with the AA or CC genotype (*P* = 0.039). (C) A trend towards an association between the MIR196A2 genotype and DFS was also observed; mean DFS was 115 months (95% CI, 99–131) for patients with the CC genotype, compared to 81 months (95% CI, 66–97) for those with the CT or TT genotype (*P* = 0.07). (D) Mean OS for 71 patients (54.2%) with the XPO5 AC genotype was 135.3 months (95% CI, 127–143) vs. 114.2 months (95% CI, 99–129) for those with the AA or CC genotype (*P* = 0.033).

Mean DFS was 106.6 months (95% CI, 96.2–117.1), and median DFS was not reached. No clinical characteristics were associated with DFS (Table 1). Of the eight miR-SNPs analyzed, only TRBP and XPO5 genotypes were associated with DFS. Mean DFS for 37 patients (31.6%) with the TRBP CC genotype was 124 months (95% CI, 112–136) vs. 86.8 months (95% CI, 74–89) for those with the TT or TC genotype (*P* = 0.022) (Figure 1A). Mean DFS for 62 patients (56.3%) with the XPO5 AC genotype was 114.2 months (95% CI, 101–127) vs. 85.8 months (95% CI, 68–104) for patients with the AA or CC genotype (*P* = 0.039) (Figure 1B). A trend towards an association between the MIR196A2 genotype and DFS was also observed; mean DFS was 115 months (95% CI, 99–131) for patients with the CC genotype, compared to 81 months (95% CI, 66–97) for those with the CT or TT genotype (*P* = 0.07) (Figure 1C).

Mean OS was 126.6 months (95% CI, 118.6–134.6), and median OS was not reached. Older age (*P*<0.001), histology other than nodular sclerosis (*P* = 0.001), B-symptoms (*P*<0.001), anemia (*P*<0.001), lymphocytopenia (*P* = 0.003), hypoalbuminemia (*P*<0.001), high LDH level (*P* = 0.021), high B-2-microglobulin level (*P*<0.001), and advanced stage (*P*<0.001) were associated with shorter OS (Table 1). Of the eight miR-SNPs analyzed, only XPO5 was associated with OS. Mean OS for 71 patients (54.2%) with the XPO5 AC genotype was 135.3 months (95% CI, 127–143) vs. 114.2 months (95% CI, 99–129) for those with the AA or CC genotype (*P* = 0.033) (Figure 1D). Table S2 displays the clinical characteristics of the HL patients stratified according to the miR-SNPs analyzed.

Since prognosis in HL has been shown to differ between patients<45 years old and those≥45years old, we performed an age-adjusted analysis of all miR-SNPs that showed significant differences in the entire cohort. The effect of TRBP on DFS that was observed in the entire cohort was maintained in both age groups (Figure 1, Figure S1). In contrast, the effect of XPO5 and MIR196A2 on DFS was maintained only in younger patients. Finally, the benefit in OS observed in the entire cohort for patients heterozygous for XPO5 was also observed in both groups in the age-adjusted analysis. (Table S3 and Figure S1).

### miR-SNPs in early- and advanced-stage HL

Since treatment strategies in HL are different for early-stage (Ann Arbor stage I and II) and advanced-stage (stage III and IV) patients [38], we examined DFS and OS according to miR-SNPs in each of these subgroups.

**Figure 2:**
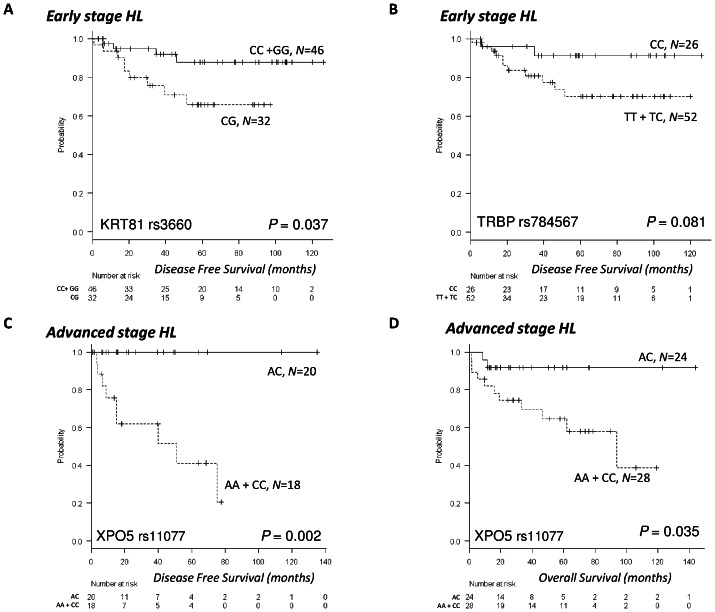
Clinical outcomes in early and advanced HL according to miR-SNPs. DFS in early-stage HL patients according to KRT81 and TRBP and in advanced HL patients according to XPO5. And OS in advanced-stage HL patients according to XPO5. (A) DFS was 72.3 months (95% CI, 59–86) for patients with the KRT81 CG genotype and 114.3 months (95% CI, 103–125) for those with the TT or TC genotype (*P* = 0.037). (B) A trend towards an association between the TRBP genotype and DFS in early-stage patients was also observed; mean DFS was 117 months (95% CI, 105–129) for patients with the TT genotype, compared to 92 months (95% CI, 78–106) for those with the CC or TC genotype (*P* = 0.081). (C) All patients with the XPO5 AC genotype were disease-free at the time of this analysis, while mean DFS among patients with the AA or CC genotype was 30.9 months (95% CI, 18–44) (*P* = 0.002). (D) OS was 133 months (95% CI, 118–148) in patients harboring the AC genotype compared to 74 months (95% CI, 55–94) for those with the AA or CC genotype (*P* = 0.035).

Mean DFS for early-stage patients was 104.6 months (95% CI, 94–115), and mean OS was 138.9 months (95% CI, 134.2–143.7). DFS was 72.3 months (95% CI, 59–86) for patients with the KRT81 CG genotype and 114.3 months (95% CI, 103–125) for those with the TT or TC genotype (*P* = 0.037) (Figure 2A). A trend towards an association between the TRBP genotype and DFS in early-stage patients was also observed; mean DFS was 117 months (95% CI, 105–129) for patients with the TT genotype, compared to 92 months (95% CI, 78–106) for those with the CC or TC genotype (*P* = 0.081) (Figure 2B). Only two deaths occurred among early-stage patients.

Mean DFS for advanced patients was 91.6 months (95%CI, 67.9–115.3), and mean OS was 101.4 months (95% CI, 82.2–120.5). Only XPO5 was associated with DFS and OS. All patients with the XPO5 AC genotype were disease-free at the time of this analysis, while mean DFS among patients with the AA or CC genotype was 30.9 months (95% CI, 18–44) (*P* = 0.002) (Figure 2C). OS was 133 months (95% CI, 118–148) in patients harboring the AC genotype compared to 74 months (95% CI, 55–94) for those with the AA or CC genotype (*P* = 0.035) (Figure 2D). When the false discovery rate was used to correct for multiple comparisons, XPO5 maintained statistical significance (adjusted *P* = 0.018).

### TRBP and XPO5 miR-SNPs in combination

**Figure 3:**
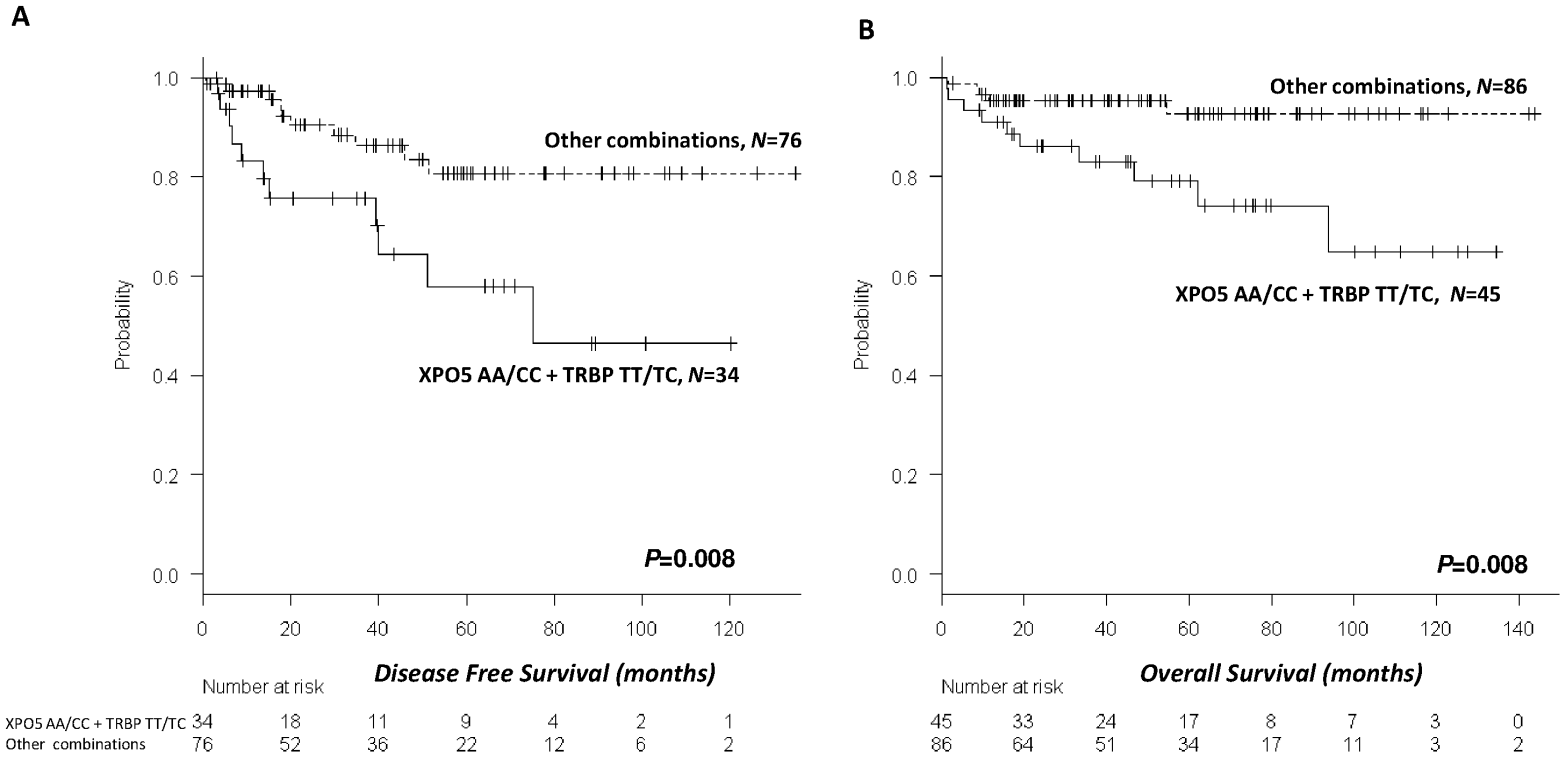
DFS and OS according to the combination of TRBP and XPO5 genotypes. Patients with both the XPO5 AA/CC and TRBP TT/TC genotypes had the worst prognosis. (A) DFS was 74 months (95% CI, 54–94) for patients with the unfavorable combination, compared to 114 months (95% CI, 102–126) for those with other combinations (*P* = 0.008). (B) OS was 103 months (95% CI, 86–120) for patients with the unfavorable combination and 135 months (95% CI, 128–143) for those with other combinations (*P* = 0.008).

Given the evidence for the influence of TRBP and XPO5 as individual markers, we then investigated the combined effect of these miR-SNPs on DFS and OS. We found a significant correlation between the TRBP/XPO5 combination and both DFS and OS. Patients with both the XPO5 AA/CC and TRBP TT/TC genotypes had the worst prognosis. DFS was 74 months (95% CI, 54–94) for patients with the unfavorable combination, compared to 114 months (95% CI, 102–126) for those with other combinations (*P* = 0.008) (Figure 3A), while OS was 103 months (95% CI, 86–120) for patients with the unfavorable combination and 135 months (95% CI, 128–143) for those with other combinations (*P* = 0.008) (Figure 3B). When the false discovery rate was used to correct for multiple comparisons, the TRBP/XPO5 combination maintained statistical significance (DFS, adjusted *P* = 0.064; OS, adjusted *P* = 0.032).

### Multivariate analyses

We performed independent multivariate analyses for DFS and OS including all variables with a univariate *P*<0.2. In the analysis for DFS, the XPO5 AA+CC genotype emerged as an independent prognostic factor (HR, 2.622; 95%CI, 1.039–6.620; *P* = 0.041), and we observed a trend towards significance for TRBP (*P* = 0.056) (Table 4).

**Table 4:** Multivariate analyses of DFS and OS.

		*P*	Hazard Ratio	95% CI
*DFS*				
	Anemia	0.441	1.4	0.5–4.2
	Age<45	0.241	0.5	0.2–1.4
	Advanced Stage	0.950	1.035	0.3–3.0
	MIR196A2 CC	0.108	0.4	0.1–1.1
	TRBP TT+TC	0.056	3.427	0.971–12.092
	**XPO5 AA+CC**	**0.041**	**2.622**	**1.039–6.620**
				
***OS***				
	B symptoms	0.562	2.3	0.1–39.7
	Nodular sclerosis subtype	0.237	0.14	0.05–3.6
	Lymphocytopenia,<0.6×10^9^/L or<8% of WBC	0.468	2.1	0.3–13.9
	High B-2-microglobulin level,>25 mg/L	0.299	3.6	0.3–40.2
	**Age**<**45**	**0.003**	**0.001**	**0.000–0.08**
	**Anemia**	**0.028**	**14.2**	**1.3–151.4**
	**Hypoalbuminemia**	**0.036**	**24.4**	**1.2–480.9**
	High LDH levels	0.052	15.2	0.98–235.7
	Advanced Stage	0.246	4.382	0.4–53.2
	EBV	0.642	1.7	0.2–16.5
	XPO5 AA+CC	0.070	5.01	0.9–28.7

In the multivariate analysis for OS, only age<45 (HR, 0.004; 95%CI, 0.000–0.109; *P* = 0.001), anemia (HR, 11.4; 95%CI, 1.447–90.683; *P* = 0.021) and hypoalbuminemia (HR, 24.4; 95%CI, 1.2–480.9; *P* = 0.036) were independent prognostic factors. We also observed a trend towards significance for high LDH (*P* = 0.052), and XPO5 genotype (*P* = 0.070) (Table 4).

## Discussion

Since the importance of miRNAs in tumorogenesis was first recognized, miRNA pharmacogenomics has emerged as a promising field [39]. The role of miR-SNPs in drug resistance, toxicity and disease progression is becoming clearer, and miR-SNPs are starting to be recognized as powerful tools for disease prognosis and diagnosis [40]. In the present study, we have analyzed the effect of eight miR-SNPs on toxicity, response, DFS and OS in 141 adult patients diagnosed with HL and found that miR-SNPs in XPO5 and TRBP – both individually and in combination – are associated with clinical outcome.

This is an exploratory study of miR-SNPs in HL, and multiple testing adjustment was not included in the original study design. When and how multiple testing adjustment should be performed is a controversial issue[41], and moreover, all the results presented here should be validated in an independent set of patients. However, in an exploratory analysis, false discovery rate adjustment was applied, and all the significant clinical characteristics shown in Table 1, as well as XPO5 in advanced-stage patients and the XPO5/TRBP combination, maintained statistical significance.

After performing the miR-SNP analyses, we observed that MIR423 (p = 0.031) and KRT81 (p = 0.041) showed differences in frequency in comparison with the European population. Both of these miR-SNPs have been reported to be related to cancer risk when compared between normal tissue of control samples and samples from cancer patients, and our findings support this. Moreover, when we analyzed if the miR-SNPs analyzed were in Hardy-Weinberg equilibrium (HWE), we found that one of them, MIR149, was not (p<0.001). Thus, it seems that an HL population is affected in some way and has more homozygotes for the SNP genotype (TT) with a decreased number of heterozygotes (CT). HWE is a mathematical construct describing a hypothetical ideal population and natural populations can sometimes approximate equilibrium but rarely precisely achieve it. Moreover, we have to take into account that we did not analyze the SNPs in normal tissue, which could partially explain these differences. Importantly, however, we have observed for the first time in HL that patients carrying the XPO5 AC genotype had both higher response rates and longer DFS and OS. These results are along the lines of previous findings in non-small-cell lung cancer [27], colorectal cancer [42], and multiple myeloma [29] where the XPO5 AC genotype was associated with better prognosis (AC+CC genotype) in NSCLC and multiple myeloma and better treatment response (AC+AA genotype) in colorectal cancer.

A reduced risk for patients with the heterozygous genotype seems difficult to explain since outcomes in heterozygous patients are usually similar to those in one of the homozygous groups (WT or SNP). However, prognosis in HL patients is usually relatively good, and consequently there are relatively few events. For this reason, a large patient cohort is needed to determine which of the homozygous groups would have as good a prognosis as the heterozygous group. When we performed an age-adjusted analysis (Table S3, Figure S1), we observed that in patients<45 years old, the WT and the heterozygous groups had the best prognosis, while in patients≥45 years old, there were clear differences in prognosis between the WT and heterozygous groups and the heterozygous group had the best prognosis in both age groups. Nevertheless, since this is an exploratory study, no clear conclusions can be drawn without further study to validate our findings.

XPO5 is the RAN-GTP-dependent protein responsible for transportation of pre-miRNA from the nucleus to the cytoplasm, and XPO5 down-regulation results in reduced miRNA levels [43]. Mutations in XPO5 have been related to reduced miRNA processing and decreased miRNA-target inhibition in several tumors [44]. However, the exact role of XPO5 dysregulation is not clear, since XPO5 is downregulated in low-grade lung adenocarcinoma [45] but upregulated in high-grade prostate cancer [46]. The miR-SNP rs11077 is located in the 3′UTR region of the XPO5 mRNA and may affect XPO5 protein levels. Patients carrying the TRBP CC genotype also had longer DFS. The TRBP rs784567 miR-SNP has previously been related to an increased risk of bladder cancer (CC genotype) [47] and oral premalignant lesions (TT genotype) [48]. TRBP plays its role in the cytoplasm, where it binds to Dicer and Ago proteins to conform the RISC complex that contributes to miRNA-mediated inhibition of translation [49]. TRBP mutations have been described in several tumors [50] and related to reduced TRBP protein expression and defective processing of miRNAs.

When we investigated the combined effect of the XPO5 and TRBP miR-SNPs, we found that patients with both the XPO5 AA/CC and TRBP TT/TC genotypes had the worst prognosis for both DFS and OS. Both XPO5 and TRBP are key players in miRNA biogenesis. Although XPO5 is located in the nuclear membrane while TRBP is located in the cytoplasm, they both interact with Dicer [51], [52], a key component in the miRNA pathway. Dicer is responsible for the cleavage of the pre-miRNA to the miRNA/miRNA* duplex [53]. XPO5 is able to mediate the nuclear export of Dicer mRNA [51] and TRBP is necessary for the recruitment of Dicer to RISC [52], suggesting that miR-SNPs in either or both of these genes may well interfere in Dicer-mediated miRNA biogenesis.

In the present study, early-stage patients carrying the KRT81 CG genotype had shorter DFS, while advanced patients carrying the XPO5 AC genotype had longer DFS and OS. Treatment decisions in HL are based in large part on disease stage at the time of diagnosis. In general, early-stage HL patients are more likely to be cured and less likely to relapse than advanced patients. However, relapse in early-stage patients is not uncommon [38], and the early identification of patients more likely to relapse could allow them to be treated with more aggressive therapies normally reserved for advanced HL. At the same time, the identification of good-prognosis patients with advanced HL could allow a reduction in intensity of chemotherapy and/or radiotherapy in these patients.

Treatment strategies in HL are often related to pulmonary and neurologic toxicity. Bleomycin-related pulmonary toxicity has been widely described in HL patients treated with ABVD or MOPPABVD [54]. Suggested risk factors for pulmonary toxicity include advanced age, bleomycin treatment, higher bleomycin dose, renal insufficiency, radiation, underlying lung disease, smoking history, and granulocyte colony-stimulating factor support [55]. We have shown here that the XPO5 AA or CC genotypes are also associated with an increased risk of bleomycin-associated pulmonary toxicity. One of the most frequent neurologic toxicities in HL is the mixed motor-sensory neuropathy associated with vincristine [56]. In the present study, the KRT81 GG genotype identified patients with an increased risk of neurological toxicity. Recent advances in the understanding of HL pathogenesis have led to the development of novel therapies targeting the microenvironment and the specific molecular pathways in HRS cells, including for example monoclonal antibodies (rituximab and alemtuzumab) [57], bortezomib or histone deacetylase inhibitors [58]. These new drugs have fewer side effects than conventional chemotherapy, and the molecular identification of patients at high risk of pulmonary or neurologic toxicity could make them candidates for treatment with these new, better-tolerated therapies.

In conclusion, miR-SNPs are a novel class of SNPs that can add useful prognostic information on the clinical outcome of HL, specifically in the identification of patients less likely to respond and more likely to relapse to standard treatments and of those at higher risk of suffering treatment-related toxicities. Importantly, the TRBP/XPO5 haplotype has surfaced as a promising prognostic factor that warrants further investigation to confirm its role as a biomarker in HL.

## Design and Methods

### Study population and ethics statement

One hundred and forty-one adult patients diagnosed with HL at Hospital Clinic in Barcelona, Spain between September 1995 and June 2005 were included in the study. Patients with available tumor samples−81% of HL patients diagnosed and treated in the center – were selected consecutively over time. The selected patients were treated by different physicians based on the common treatment criteria of the Hematology Department of Hospital Clinic, Barcelona.

The clinical parameters included in this study are internationally accepted as relevant in HL and are included in international prognostic indexes [30], [31]. Anemia, leukocytosis, lymphopenia, albuminemia were determined by standard blood analyses. B symptoms, the standard method for classifying lymphomas according to clinical symptoms, include fever, night sweats and weight loss of>10% of total body weight over the last 6 months. Bulky mass is defined as>1/3 widening or mediastinum at T5-6, or>10 cm dimension of nodal mass. B2M (beta 2 microglobulin) was determined by immunonephelometry using reagents from Siemens Healthcare (Germany) and automatically analyzed with the Siemens BNII system. LDH (lactate dehydrogenase) was analyzed with the Advia Chemistry System (Siemens). The presence of EBV in HL lymph nodes was examined by in situ hybridization for EBV RNA in an automated platform BenchMark XT (EBER 1 and 2, Inform EBER; Ventana Medical Systems, Tucson, AZ). Toxicities were determined according to the Common Toxicity Criteria (CTC) of the EORTC. Approval for the study was obtained from the Clinical Research Ethics Committee of the Hospital Clínic de Barcelona (CEIC Hospital Clínic), and written informed consent was obtained from each participant in accordance with the Declaration of Helsinki.

### Selection of the miR-SNPs

In two previous studies by our group[27], [29], we found that some miR-SNPs had prognostic implications in non-small-cell lung cancer and multiple myeloma. Based on these previous findings, we have analyzed eight miR-SNPs in genes involved in miRNA regulatory pathways: four in miRNA genes (MIR196A2 rs11614913; MIR149 rs2292832; MIR423 rs6505162; MIR146 rs2910164); two in miRNA binding sites in the keratin 81 (KRT81 rs3660) and family with sequence similarity 179, member B (FAM179B rs1053667); and three in the miRNA-processing machinery genes exportin 5 (XPO5 rs11077) and TAR RNA binding protein 2 (TRBP rs784567). All SNPs were previously selected according to the following criteria: firstly, a determined allele frequency for the European population and availability in the National Center for Biotechnology Information (NCBI) SNP database; secondly, a genotype frequency for the European population≥0.05; and finally, either a known association with a differential susceptibility to cancer development or clinical outcome in other tumors. The two SNPs in miRNA binding sites had previously been reported to have an aberrant allelic frequency in human tumors [32]. Moreover, three of the eight SNPs have been shown to have functional implications, as demonstrated by our group (XPO5, KRT81)[27], [29] and others (MIR196A2)[33].

### DNA extraction and genotyping

DNA was obtained from formalin-fixed, paraffin-embedded lymph nodes using the commercial DNeasy tissue kit (Qiagen, Valencia, CA) following the manufacturer's protocol. DNA was quantified with a NanoDrop ND-1000 spectrophotometer (Thermo Fisher Scientific Inc., Waltham, MA). SNP analysis was performed by allelic discrimination on ABI Prism 7500 as previously described [27]. Primers and probes were commercially available (TaqMan SNP Genotyping Assays, Applied Biosystems, Foster City, CA).

### Statistical analyses

The present work is retrospective analysis of miR-SNPS in HL patients. The analysis is based on a median follow up of 50 months. All the clinical characteristics were obtained at diagnosis. The two primary endpoints analyzed were disease-free survival (DFS) and overall survival (OS). DFS was measured from the time of occurrence of a disease-free state or attainment of a complete response (CR) to disease recurrence or death as a result of lymphoma or acute toxicity of treatment. OS was calculated from the time of diagnosis to the date of death or last follow-up. The Kaplan –Meier method was used to estimate DFS and OS, and comparison between risk groups was performed by using the log-rank test[34], [35]. A multivariate regression analysis assessing the significance of individual clinical factors included in the International Score (all prognostic variables in the univariate analysis with a *P*-value less than or equal to 0.2) and significant miR-SNPs was performed by using the Cox proportional hazards model with backward selection[36]. The proportional hazard assumption was tested for each variable by analyzing the Schoenfeld residuals(R software). As secondary endpoints, we analyzed the association of miR-SNPs with treatment-related toxicities and treatment response. The Chi-squared or Fisher's exact test was used to estimate differences in distributions. The multivariate analysis for toxicity was performed by using Binary Logistic regression. All statistical analyses were performed using PAS W Statistics 18 (SPSS Inc., Chicago, IL) and R software (v2.13.2). The level of significance was set at≤0.05. The statistical power of the study, calculated using GWAPower[37], was 0.81580 with the mean sample size of n = 125 (range 0.74561–0.85541).

## Supporting Information

Figure S1 **Age adjusted analysis (Age<45 and age≥45).** The statistical power of the age adjusted analysis, calculated using GWAPower, was 0.77014 for the Age<45 subgroup with the sample size of n = 110, and it was 0.34112 for the Age≥45 subgroup with the sample size of n = 31.
(DOCX)

Table S1 **Univarite analysis of the association between treatment-related toxicities and main clinical characteristics.**
(DOCX)

Table S2 **Clinical characteristics of HL patients stratified according to miRNA-SNPs.** EBV status was only available for 105 patients.
(DOCX)

Table S3 **Age adjusted analysis (Age<45 and age≥45).**
(DOCX)
