# Supplementary material for: MiR-SNPs as Markers of Toxicity and Clinical Outcome in Hodgkin Lymphoma Patients
Source: PLoS One. 2013 May 21;8(5):e64716. doi: 10.1371/journal.pone.0064716 (PMC3660374; doi:10.1371/journal.pone.0064716)
Supplement: Figure S1 — Age adjusted analysis (Age<45 and age≥45). The statistical power of the age adjusted analysis, calculated using GWAPower, was 0.77014 for the Age<45 subgroup with the sample size of n = 110, and it was 0.34112 for the Age≥45 subgroup with the sample size of n = 31. (DOCX) [file pone.0064716.s001.docx]

**Figure S1.** Age adjusted analysis (Age<45 and age≥45). The statistical power of the age adjusted analysis, calculated using GWAPower, was 0.77014 for the Age<45 subgroup with the sample size of n=110, and it was 0.34112 for the Age≥45 subgroup with the sample size of n=31.

1. **TREATMENT RELATED TOXICITIES AGE ADJUSTED ANALYSIS**

In the age adjusted analysis for treatment related toxicities, the KRT81 and the XPO5 retained their significance in the Age<45 group, where patients harboring the KRT81 GG genotype had a higher rate of neurological toxicity than those with the CC or CG genotype (36% vs. 12%; P = 0.014). Patients carrying the XPO5 AA or CC genotype had a higher incidence of bleomycin-associated pulmonary toxicity than those with the AC genotype (11% vs. 0%; P = 0.019). While in the age≥45 group no significant differences where observed (KRT81: 14% vs. 11%; P = 1.0; XPO5: 7% vs. 6%; P = 1.0).

1. **DFS and OS AGE ADJUSTED ANALYSIS**

| **DFS AGE<45 ADJUSTED ANALYSIS** |  |
| --- | --- |
| TRBP, p=0.096 | XPO5, p=0.043 |
| 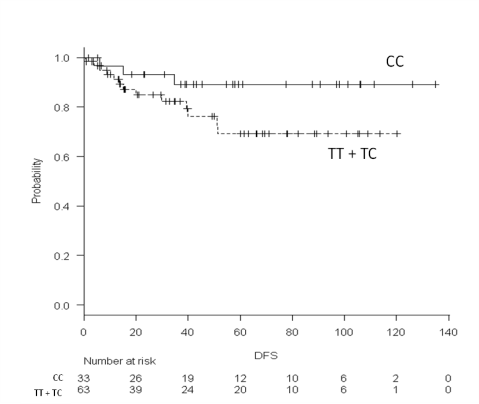 | 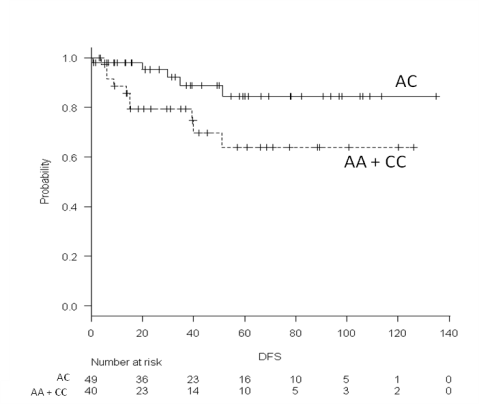 |
| MIR196A2, p=0.066 | MIR196A2, p=0.039 |
| 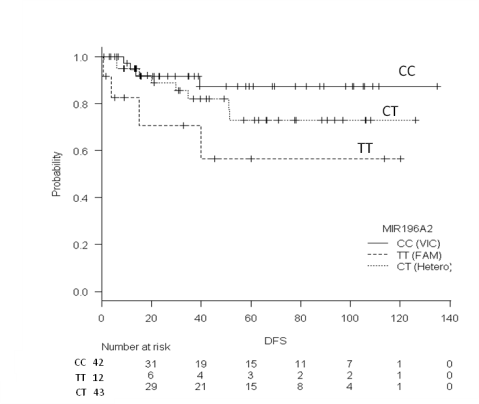 | 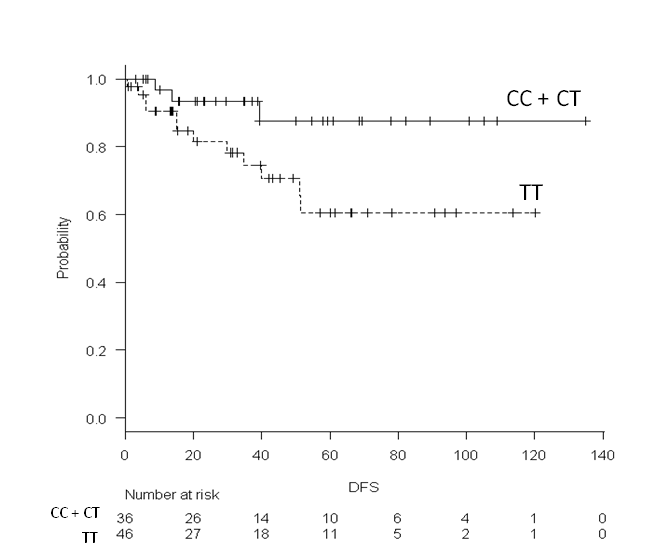 |
|  |  |
| **DFS AGE≥45 ADJUSTED ANALYSIS** |  |
| TRBP, p=0.161 | XPO5, p=0.551 |
| **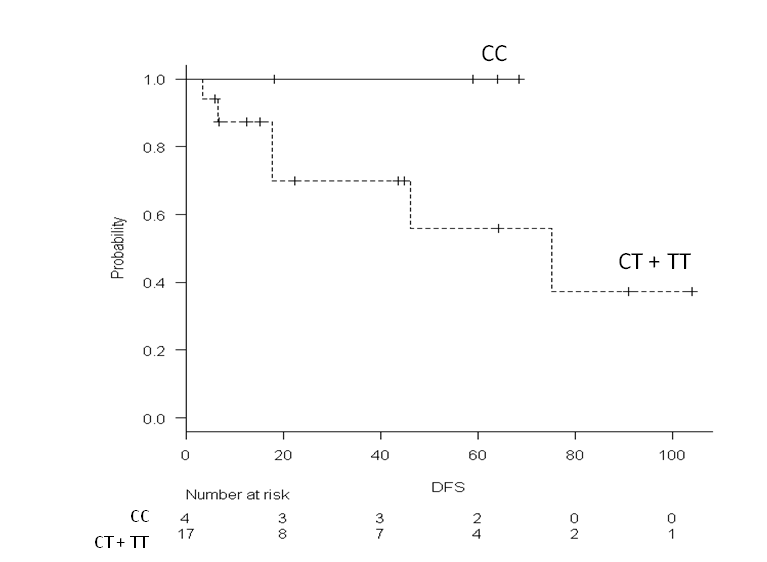** | **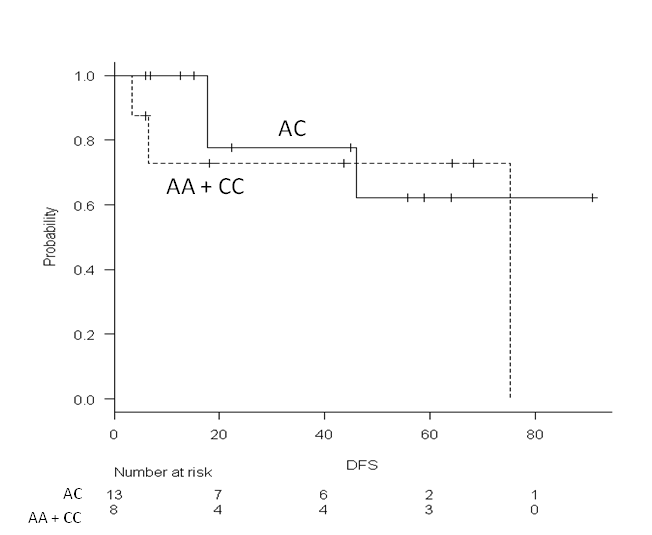** |
| MIR196A2, p=0.831 |  |
| 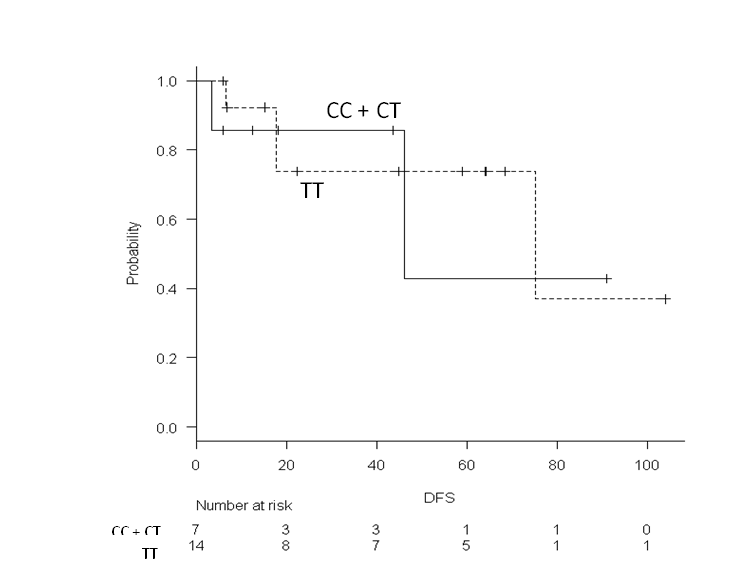 |  |

| **OS AGE<45 ADJUSTED ANALYSIS** |  |
| --- | --- |
| XPO5, p=0.064 | XPO5, p= 0.262 |
| 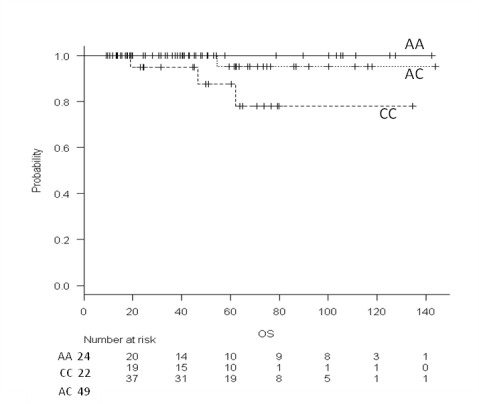 | 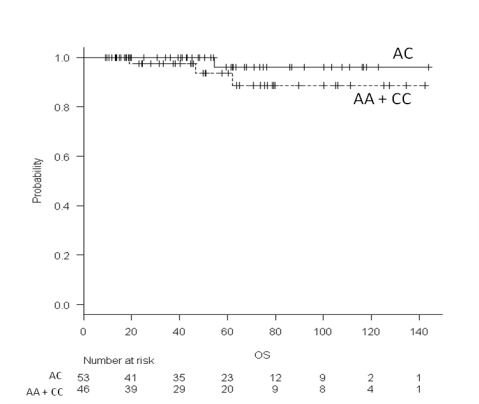 |
|  |  |
| **OS AGE≥45 ADJUSTED ANALYSIS** |  |
| XPO5, p=0.045 | XPO5, p=0.015 |
| 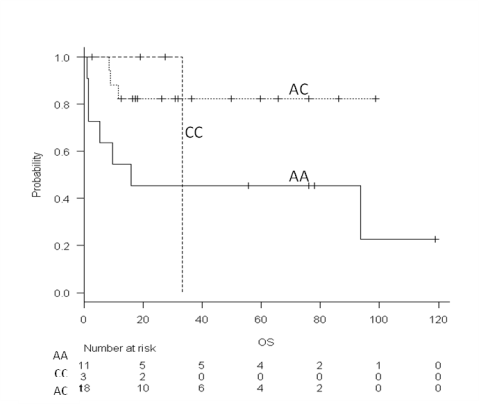 | 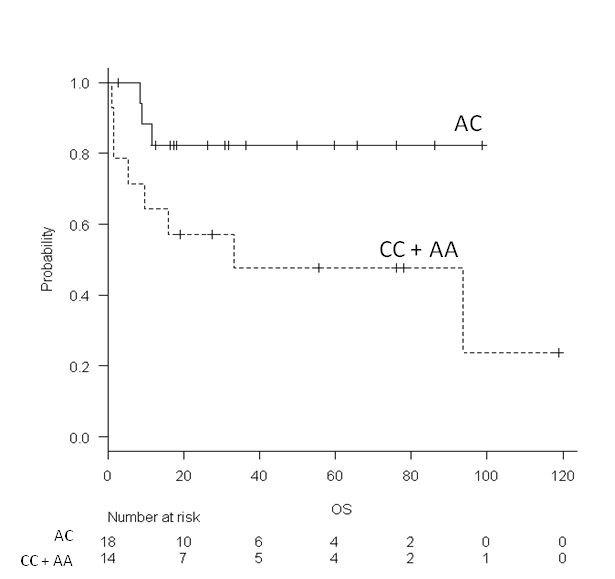 |
