# Supplementary material for: MiR-SNPs as Markers of Toxicity and Clinical Outcome in Hodgkin Lymphoma Patients
Source: PLoS One. 2013 May 21;8(5):e64716. doi: 10.1371/journal.pone.0064716 (PMC3660374; doi:10.1371/journal.pone.0064716)
Supplement: Table S1 — Univarite analysis of the association between treatment-related toxicities and main clinical characteristics. (DOCX) [file pone.0064716.s002.docx]

**Table S1.** Univarite analysis of the association between treatment-related toxicities and main clinical characteristics.

| **Characteristic** | ***Neutropenia***  ***p-value*** | ***Anemia***  ***p-value*** | ***Thrombocytopenia***  ***p-value*** | ***Pulmonary toxicity***  ***p-value*** | ***Neurological toxicity***  ***p-value*** | ***Infectious toxicity***  ***p-value*** |
| --- | --- | --- | --- | --- | --- | --- |
| **Sex** | 0.159 | 0.111 | 0.111 | 0.265 | 0.173 | 0.166 |
| **Age ≥45** | 0.133 | **p<0.001** | **0.021** | 0.642 | 0.409 | 0.291 |
| **Histology** **(Nodular sclerosis vs Other)** | 0.717 | 0.081 | 0.690 | 0.443 | 0.355 | 0.593 |
| **B symptoms** | 0.149 | 0.083 | 0.240 | 0.451 | 0.820 | 0.220 |
| **Bulky mass** | 1.00 | 0.346 | 0.346 | 1.00 | 1.00 | 0.665 |
| **Anemia, Hb levels less than 10^5^ g/L** | **0.018** | **0.002** | 0.126 | 0.169 | 0.409 | 0.291 |
| **Leukocytosis, more than 15X10^9^/L** | 0.422 | 1.00 | 1.00 | 1.00 | 0.306 | 0.600 |
| **Lymphocytopenia, <0.6 X 10^9^/L or <8% of WBC** | 0.094 | 0.159 | 0.159 | 1.00 | 0.485 | 0.174 |
| **Hypoalbuminemia, <40 g/L** | 0.564 | 0.182 | 0.420 | 0.691 | 1.00 | 0.571 |
| **High LDH level, >450 UI/L** | 0.073 | **0.057** | **0.008** | 0.673 | 0.128 | **0.006** |
| **High B-2-microglobulin level, >25 mg/L** | **0.002** | **0.001** | **0.044** | 0.153 | 1.00 | **0.001** |
| **Advanced Stage** | 0.098 | **0.032** | 0.205 | 0.207 | 0.354 | **0.030** |
| **EBV** | **0.013** | 0.068 | 0.068 | 0.669 | **0.029** | 0.063 |
