# Supplementary material for: MiR-SNPs as Markers of Toxicity and Clinical Outcome in Hodgkin Lymphoma Patients
Source: PLoS One. 2013 May 21;8(5):e64716. doi: 10.1371/journal.pone.0064716 (PMC3660374; doi:10.1371/journal.pone.0064716)
Supplement: Table S2 — Clinical characteristics of HL patients stratified according to miRNA-SNPs. EBV status was only available for 105 patients. (DOCX) [file pone.0064716.s003.docx]

**Table S2.** C*linical characteristics of HL patients stratified according to miRNA-SNPs. EBV status was only available for 105 patients.*

1. **MIR196A2 rs11614913 (1**🡪**CC; 2**🡪**TT; 3**🡪**CT).**

| **Variable** | **Levels** | **N_1_** | **%_1_** | **∑%1** | **N_2_** | **%_2_** | **∑%_2_** | **N_3_** | **%_3_** | **∑%_3_** | **N_all_** | **%_all_** | **∑%_all_** |
| --- | --- | --- | --- | --- | --- | --- | --- | --- | --- | --- | --- | --- | --- |
| Sex | female | 36 | 59.0 | 59.0 | 9 | 52.9 | 52.9 | 23 | 37.1 | 37.1 | 69 | 48.9 | 48.9 |
|  | male | 25 | 41.0 | 100.0 | 8 | 47.1 | 100.0 | 39 | 62.9 | 100.0 | 72 | 51.1 | 100.0 |
| p = 0.048 | all | 61 | 100.0 |  | 17 | 100.0 |  | 62 | 100.0 |  | 141 | 100.0 |  |
| Age | <45 | 48 | 78.7 | 78.7 | 12 | 70.6 | 70.6 | 50 | 80.7 | 80.7 | 110 | 78.0 | 78.0 |
|  | ≥45 | 13 | 21.3 | 100.0 | 5 | 29.4 | 100.0 | 12 | 19.4 | 100.0 | 31 | 22.0 | 100.0 |
| p = 0.669 | all | 61 | 100.0 |  | 17 | 100.0 |  | 62 | 100.0 |  | 141 | 100.0 |  |
| Histology | EN | 43 | 70.5 | 70.5 | 6 | 35.3 | 35.3 | 34 | 54.8 | 54.8 | 83 | 58.9 | 58.9 |
|  | other | 18 | 29.5 | 100.0 | 11 | 64.7 | 100.0 | 28 | 45.2 | 100.0 | 58 | 41.1 | 100.0 |
| p = 0.021 | all | 61 | 100.0 |  | 17 | 100.0 |  | 62 | 100.0 |  | 141 | 100.0 |  |
| B symptoms | No | 40 | 65.6 | 65.6 | 10 | 58.8 | 58.8 | 30 | 49.2 | 49.2 | 81 | 57.9 | 57.9 |
|  | Yes | 21 | 34.4 | 100.0 | 7 | 41.2 | 100.0 | 31 | 50.8 | 100.0 | 59 | 42.1 | 100.0 |
| p = 0.186 | all | 61 | 100.0 |  | 17 | 100.0 |  | 61 | 100.0 |  | 140 | 100.0 |  |
| Bulky mass | No | 43 | 70.5 | 70.5 | 17 | 100.0 | 100.0 | 51 | 82.3 | 82.3 | 112 | 79.4 | 79.4 |
|  | Yes | 18 | 29.5 | 100.0 | 0 | 0.0 | 100.0 | 11 | 17.7 | 100.0 | 29 | 20.6 | 100.0 |
| p = 0.022 | all | 61 | 100.0 |  | 17 | 100.0 |  | 62 | 100.0 |  | 141 | 100.0 |  |
| Anemia, Hb levels less than 10^5^ g/L | No | 49 | 80.3 | 80.3 | 11 | 64.7 | 64.7 | 49 | 79.0 | 79.0 | 110 | 78.0 | 78.0 |
|  | Yes | 12 | 19.7 | 100.0 | 6 | 35.3 | 100.0 | 13 | 21.0 | 100.0 | 31 | 22.0 | 100.0 |
| p = 0.373 | all | 61 | 100.0 |  | 17 | 100.0 |  | 62 | 100.0 |  | 141 | 100.0 |  |
| Leukocytosis, more than 15X10^9^/L | No | 49 | 81.7 | 81.7 | 16 | 94.1 | 94.1 | 57 | 91.9 | 91.9 | 123 | 87.9 | 87.9 |
|  | Yes | 11 | 18.3 | 100.0 | 1 | 5.9 | 100.0 | 5 | 8.1 | 100.0 | 17 | 12.1 | 100.0 |
| p = 0.155 | all | 60 | 100.0 |  | 17 | 100.0 |  | 62 | 100.0 |  | 140 | 100.0 |  |
| Lymphocytopenia, <0.6 X 10^9^/L or <8% of WBC | No | 56 | 93.3 | 93.3 | 16 | 100.0 | 100.0 | 48 | 78.7 | 78.7 | 121 | 87.7 | 87.7 |
|  | Yes | 4 | 6.7 | 100.0 | 0 | 0.0 | 100.0 | 13 | 21.3 | 100.0 | 17 | 12.3 | 100.0 |
| p = 0.015 | all | 60 | 100.0 |  | 16 | 100.0 |  | 61 | 100.0 |  | 138 | 100.0 |  |
| Hypoalbuminemia, <40 g/L | No | 43 | 74.1 | 74.1 | 10 | 62.5 | 62.5 | 32 | 56.1 | 56.1 | 86 | 65.2 | 65.2 |
|  | Yes | 15 | 25.9 | 100.0 | 6 | 37.5 | 100.0 | 25 | 43.9 | 100.0 | 46 | 34.9 | 100.0 |
| p = 0.127 | all | 58 | 100.0 |  | 16 | 100.0 |  | 57 | 100.0 |  | 132 | 100.0 |  |
| High LDH level, >450 UI/L | No | 42 | 70.0 | 70.0 | 10 | 62.5 | 62.5 | 46 | 74.2 | 74.2 | 99 | 71.2 | 71.2 |
|  | Yes | 18 | 30.0 | 100.0 | 6 | 37.5 | 100.0 | 16 | 25.8 | 100.0 | 40 | 28.8 | 100.0 |
| p = 0.638 | all | 60 | 100.0 |  | 16 | 100.0 |  | 62 | 100.0 |  | 139 | 100.0 |  |
| High B-2-microglobulin level, >25 mg/L | No | 35 | 79.5 | 79.5 | 9 | 64.3 | 64.3 | 39 | 75.0 | 75.0 | 84 | 75.7 | 75.7 |
|  | Yes | 9 | 20.4 | 100.0 | 5 | 35.7 | 100.0 | 13 | 25.0 | 100.0 | 27 | 24.3 | 100.0 |
| p = 0.510 | all | 44 | 100.0 |  | 14 | 100.0 |  | 52 | 100.0 |  | 111 | 100.0 |  |
| Stage | Early(I‐II) | 39 | 63.9 | 63.9 | 8 | 47.1 | 47.1 | 38 | 62.3 | 62.3 | 86 | 61.4 | 61.4 |
|  | Advanced (III‐IV) | 22 | 36.1 | 100.0 | 9 | 52.9 | 100.0 | 23 | 37.7 | 100.0 | 54 | 38.6 | 100.0 |
| p = 0.437 | all | 61 | 100.0 |  | 17 | 100.0 |  | 61 | 100.0 |  | 140 | 100.0 |  |
| Treatment | ABVD | 32 | 52.5 | 52.5 | 10 | 62.5 | 62.5 | 30 | 50.0 | 50.0 | 73 | 52.9 | 52.9 |
|  | MOPABV | 25 | 41.0 | 93.4 | 3 | 18.8 | 81.2 | 25 | 41.7 | 91.7 | 53 | 38.4 | 91.3 |
|  | MOPP | 3 | 4.9 | 98.4 | 2 | 12.5 | 93.8 | 3 | 5.0 | 96.7 | 8 | 5.8 | 97.1 |
|  | Other | 1 | 1.6 | 100.0 | 1 | 6.2 | 100.0 | 2 | 3.3 | 100.0 | 4 | 2.9 | 100.0 |
| p = 0.596 | all | 61 | 100.0 |  | 16 | 100.0 |  | 60 | 100.0 |  | 138 | 100.0 |  |
| EBV | No | 31 | 67.4 | 67.4 | 9 | 60.0 | 60.0 | 24 | 55.8 | 55.8 | 65 | 61.9 | 61.9 |
|  | Yes | 15 | 32.6 | 100.0 | 6 | 40.0 | 100.0 | 19 | 44.2 | 100.0 | 40 | 38.1 | 100.0 |
| p = 0.528 | all | 46 | 100.0 |  | 15 | 100.0 |  | 43 | 100.0 |  | 105 | 100.0 |  |
| Neutropenia toxicity | No | 38 | 62.3 | 62.3 | 8 | 47.1 | 47.1 | 47 | 75.8 | 75.8 | 94 | 66.7 | 66.7 |
|  | Yes | 23 | 37.7 | 100.0 | 9 | 52.9 | 100.0 | 15 | 24.2 | 100.0 | 47 | 33.3 | 100.0 |
| p = 0.056 | all | 61 | 100.0 |  | 17 | 100.0 |  | 62 | 100.0 |  | 141 | 100.0 |  |
| Anemia toxicity | No | 58 | 95.1 | 95.1 | 15 | 88.2 | 88.2 | 61 | 98.4 | 98.4 | 135 | 95.7 | 95.7 |
|  | Yes | 3 | 4.9 | 100.0 | 2 | 11.8 | 100.0 | 1 | 1.6 | 100.0 | 6 | 4.3 | 100.0 |
| p = 0.177 | all | 61 | 100.0 |  | 17 | 100.0 |  | 62 | 100.0 |  | 141 | 100.0 |  |
| Thrombocytopenia toxicity | No | 57 | 93.4 | 93.4 | 17 | 100.0 | 100.0 | 60 | 96.8 | 96.8 | 135 | 95.7 | 95.7 |
|  | Yes | 4 | 6.6 | 100.0 | 0 | 0.0 | 100.0 | 2 | 3.2 | 100.0 | 6 | 4.3 | 100.0 |
| p = 0.428 | all | 61 | 100.0 |  | 17 | 100.0 |  | 62 | 100.0 |  | 141 | 100.0 |  |
| Pulmonary toxicity | No | 60 | 98.4 | 98.4 | 16 | 94.1 | 94.1 | 56 | 91.8 | 91.8 | 133 | 95.0 | 95.0 |
|  | Yes | 1 | 1.6 | 100.0 | 1 | 5.9 | 100.0 | 5 | 8.2 | 100.0 | 7 | 5.0 | 100.0 |
| p = 0.250 | all | 61 | 100.0 |  | 17 | 100.0 |  | 61 | 100.0 |  | 140 | 100.0 |  |
| Neurological toxicity | No | 51 | 83.6 | 83.6 | 16 | 94.1 | 94.1 | 50 | 80.7 | 80.7 | 118 | 83.7 | 83.7 |
|  | Yes | 10 | 16.4 | 100.0 | 1 | 5.9 | 100.0 | 12 | 19.4 | 100.0 | 23 | 16.3 | 100.0 |
| p = 0.414 | all | 61 | 100.0 |  | 17 | 100.0 |  | 62 | 100.0 |  | 141 | 100.0 |  |
| Infectious toxicity | No | 36 | 59.0 | 59.0 | 9 | 52.9 | 52.9 | 44 | 71.0 | 71.0 | 90 | 63.8 | 63.8 |
|  | Yes | 25 | 41.0 | 100.0 | 8 | 47.1 | 100.0 | 18 | 29.0 | 100.0 | 51 | 36.2 | 100.0 |
| p = 0.241 | all | 61 | 100.0 |  | 17 | 100.0 |  | 62 | 100.0 |  | 141 | 100.0 |  |

1. **MIR149 rs2292832 (1🡪CC; 2🡪TT; 3🡪CT).**

| **Variable** | **Levels** | **N_1_** | **%_1_** | **∑%1** | **N_2_** | **%_2_** | **∑%2** | **N_3_** | **%_3_** | **∑%_3_** | **N_all_** | **%_all_** | **∑%_all_** |
| --- | --- | --- | --- | --- | --- | --- | --- | --- | --- | --- | --- | --- | --- |
| Sex | female | 26 | 42.6 | 42.6 | 9 | 60.0 | 60.0 | 14 | 50.0 | 50.0 | 69 | 48.9 | 48.9 |
|  | male | 35 | 57.4 | 100.0 | 6 | 40.0 | 100.0 | 14 | 50.0 | 100.0 | 72 | 51.1 | 100.0 |
| p = 0.452 | all | 61 | 100.0 |  | 15 | 100.0 |  | 28 | 100.0 |  | 141 | 100.0 |  |
| Age | <45 | 43 | 70.5 | 70.5 | 12 | 80.0 | 80.0 | 24 | 85.7 | 85.7 | 110 | 78.0 | 78.0 |
|  | ≥45 | 18 | 29.5 | 100.0 | 3 | 20.0 | 100.0 | 4 | 14.3 | 100.0 | 31 | 22.0 | 100.0 |
| p = 0.274 | all | 61 | 100.0 |  | 15 | 100.0 |  | 28 | 100.0 |  | 141 | 100.0 |  |
| Histology | EN | 31 | 50.8 | 50.8 | 9 | 60.0 | 60.0 | 14 | 50.0 | 50.0 | 83 | 58.9 | 58.9 |
|  | other | 30 | 49.2 | 100.0 | 6 | 40.0 | 100.0 | 14 | 50.0 | 100.0 | 58 | 41.1 | 100.0 |
| p = 0.793 | all | 61 | 100.0 |  | 15 | 100.0 |  | 28 | 100.0 |  | 141 | 100.0 |  |
| B symptoms | No | 33 | 54.1 | 54.1 | 6 | 40.0 | 40.0 | 21 | 75.0 | 75.0 | 81 | 57.9 | 57.9 |
|  | Yes | 28 | 45.9 | 100.0 | 9 | 60.0 | 100.0 | 7 | 25.0 | 100.0 | 59 | 42.1 | 100.0 |
| p = 0.058 | all | 61 | 100.0 |  | 15 | 100.0 |  | 28 | 100.0 |  | 140 | 100.0 |  |
| Bulky mass | No | 53 | 86.9 | 86.9 | 13 | 86.7 | 86.7 | 21 | 75.0 | 75.0 | 112 | 79.4 | 79.4 |
|  | Yes | 8 | 13.1 | 100.0 | 2 | 13.3 | 100.0 | 7 | 25.0 | 100.0 | 29 | 20.6 | 100.0 |
| p = 0.350 | all | 61 | 100.0 |  | 15 | 100.0 |  | 28 | 100.0 |  | 141 | 100.0 |  |
| Anemia, Hb levels less than 10^5^ g/L | No | 50 | 82.0 | 82.0 | 8 | 53.3 | 53.3 | 23 | 82.1 | 82.1 | 110 | 78.0 | 78.0 |
|  | Yes | 11 | 18.0 | 100.0 | 7 | 46.7 | 100.0 | 5 | 17.9 | 100.0 | 31 | 22.0 | 100.0 |
| p = 0.046 | all | 61 | 100.0 |  | 15 | 100.0 |  | 28 | 100.0 |  | 141 | 100.0 |  |
| Leukocytosis, more than 15X10^9^/L | No | 52 | 86.7 | 86.7 | 14 | 93.3 | 93.3 | 23 | 82.1 | 82.1 | 123 | 87.9 | 87.9 |
|  | Yes | 8 | 13.3 | 100.0 | 1 | 6.7 | 100.0 | 5 | 17.9 | 100.0 | 17 | 12.1 | 100.0 |
| p = 0.591 | all | 60 | 100.0 |  | 15 | 100.0 |  | 28 | 100.0 |  | 140 | 100.0 |  |
| Lymphocytopenia, <0.6 X 10^9^/L or <8% of WBC | No | 52 | 88.1 | 88.1 | 11 | 73.3 | 73.3 | 25 | 92.6 | 92.6 | 121 | 87.7 | 87.7 |
|  | Yes | 7 | 11.9 | 100.0 | 4 | 26.7 | 100.0 | 2 | 7.4 | 100.0 | 17 | 12.3 | 100.0 |
| p = 0.190 | all | 59 | 100.0 |  | 15 | 100.0 |  | 27 | 100.0 |  | 138 | 100.0 |  |
| Hypoalbuminemia, <40 g/L | No | 39 | 69.6 | 69.6 | 8 | 57.1 | 57.1 | 19 | 73.1 | 73.1 | 86 | 65.2 | 65.2 |
|  | Yes | 17 | 30.4 | 100.0 | 6 | 42.9 | 100.0 | 7 | 26.9 | 100.0 | 46 | 34.9 | 100.0 |
| p = 0.569 | all | 56 | 100.0 |  | 14 | 100.0 |  | 26 | 100.0 |  | 132 | 100.0 |  |
| High LDH level, >450 UI/L | No | 45 | 73.8 | 73.8 | 8 | 53.3 | 53.3 | 18 | 69.2 | 69.2 | 99 | 71.2 | 71.2 |
|  | Yes | 16 | 26.2 | 100.0 | 7 | 46.7 | 100.0 | 8 | 30.8 | 100.0 | 40 | 28.8 | 100.0 |
| p = 0.304 | all | 61 | 100.0 |  | 15 | 100.0 |  | 26 | 100.0 |  | 139 | 100.0 |  |
| High B-2-microglobulin level, >25 mg/L | No | 36 | 73.5 | 73.5 | 8 | 61.5 | 61.5 | 13 | 72.2 | 72.2 | 84 | 75.7 | 75.7 |
|  | Yes | 13 | 26.5 | 100.0 | 5 | 38.5 | 100.0 | 5 | 27.8 | 100.0 | 27 | 24.3 | 100.0 |
| p = 0.696 | all | 49 | 100.0 |  | 13 | 100.0 |  | 18 | 100.0 |  | 111 | 100.0 |  |
| Stage | Early(I‐II) | 37 | 60.7 | 60.7 | 8 | 53.3 | 53.3 | 17 | 63.0 | 63.0 | 86 | 61.4 | 61.4 |
|  | Advanced (III‐IV) | 24 | 39.3 | 100.0 | 7 | 46.7 | 100.0 | 10 | 37.0 | 100.0 | 54 | 38.6 | 100.0 |
| p = 0.824 | all | 61 | 100.0 |  | 15 | 100.0 |  | 27 | 100.0 |  | 140 | 100.0 |  |
| Treatment | ABVD | 38 | 63.3 | 63.3 | 10 | 66.7 | 66.7 | 21 | 77.8 | 77.8 | 73 | 52.9 | 52.9 |
|  | MOPABV | 18 | 30.0 | 93.3 | 2 | 13.3 | 80.0 | 4 | 14.8 | 92.6 | 53 | 38.4 | 91.3 |
|  | MOPP | 3 | 5.0 | 98.3 | 2 | 13.3 | 93.3 | 1 | 3.7 | 96.3 | 8 | 5.8 | 97.1 |
|  | Other | 1 | 1.7 | 100.0 | 1 | 6.7 | 100.0 | 1 | 3.7 | 100.0 | 4 | 2.9 | 100.0 |
| p = 0.425 | all | 60 | 100.0 |  | 15 | 100.0 |  | 27 | 100.0 |  | 138 | 100.0 |  |
| EBV | No | 30 | 58.8 | 58.8 | 7 | 53.9 | 53.9 | 17 | 65.4 | 65.4 | 65 | 61.9 | 61.9 |
|  | Yes | 21 | 41.2 | 100.0 | 6 | 46.1 | 100.0 | 9 | 34.6 | 100.0 | 40 | 38.1 | 100.0 |
| p = 0.759 | all | 51 | 100.0 |  | 13 | 100.0 |  | 26 | 100.0 |  | 105 | 100.0 |  |
| Neutropenia toxicity | No | 37 | 60.7 | 60.7 | 10 | 66.7 | 66.7 | 17 | 60.7 | 60.7 | 94 | 66.7 | 66.7 |
|  | Yes | 24 | 39.3 | 100.0 | 5 | 33.3 | 100.0 | 11 | 39.3 | 100.0 | 47 | 33.3 | 100.0 |
| p = 0.907 | all | 61 | 100.0 |  | 15 | 100.0 |  | 28 | 100.0 |  | 141 | 100.0 |  |
| Anemia toxicity | No | 57 | 93.4 | 93.4 | 14 | 93.3 | 93.3 | 28 | 100.0 | 100.0 | 135 | 95.7 | 95.7 |
|  | Yes | 4 | 6.6 | 100.0 | 1 | 6.7 | 100.0 | 0 | 0.0 | 100.0 | 6 | 4.3 | 100.0 |
| p = 0.380 | all | 61 | 100.0 |  | 15 | 100.0 |  | 28 | 100.0 |  | 141 | 100.0 |  |
| Thrombocytopenia toxicity | No | 57 | 93.4 | 93.4 | 15 | 100.0 | 100.0 | 28 | 100.0 | 100.0 | 135 | 95.7 | 95.7 |
|  | Yes | 4 | 6.6 | 100.0 | 0 | 0.0 | 100.0 | 0 | 0.0 | 100.0 | 6 | 4.3 | 100.0 |
| p = 0.231 | all | 61 | 100.0 |  | 15 | 100.0 |  | 28 | 100.0 |  | 141 | 100.0 |  |
| Pulmonary toxicity | No | 59 | 96.7 | 96.7 | 15 | 100.0 | 100.0 | 24 | 88.9 | 88.9 | 133 | 95.0 | 95.0 |
|  | Yes | 2 | 3.3 | 100.0 | 0 | 0.0 | 100.0 | 3 | 11.1 | 100.0 | 7 | 5.0 | 100.0 |
| p = 0.184 | all | 61 | 100.0 |  | 15 | 100.0 |  | 27 | 100.0 |  | 140 | 100.0 |  |
| Neurological toxicity | No | 53 | 86.9 | 86.9 | 14 | 93.3 | 93.3 | 25 | 89.3 | 89.3 | 118 | 83.7 | 83.7 |
|  | Yes | 8 | 13.1 | 100.0 | 1 | 6.7 | 100.0 | 3 | 10.7 | 100.0 | 23 | 16.3 | 100.0 |
| p = 0.772 | all | 61 | 100.0 |  | 15 | 100.0 |  | 28 | 100.0 |  | 141 | 100.0 |  |
| Infectious toxicity | No | 38 | 62.3 | 62.3 | 10 | 66.7 | 66.7 | 17 | 60.7 | 60.7 | 90 | 63.8 | 63.8 |
|  | Yes | 23 | 37.7 | 100.0 | 5 | 33.3 | 100.0 | 11 | 39.3 | 100.0 | 51 | 36.2 | 100.0 |
| p = 0.927 | all | 61 | 100.0 |  | 15 | 100.0 |  | 28 | 100.0 |  | 141 | 100.0 |  |

1. **MIR423 rs6505162 (1🡪CC; 2🡪AA; 3🡪AC).**

| **Variable** | **Levels** | **N_1_** | **%_1_** | **∑%_1_** | **N_2_** | **%_2_** | **∑%_2_** | **N_3_** | **%_3_** | **∑%_3_** | **N_all_** | **%_all_** | **∑%_all_** |
| --- | --- | --- | --- | --- | --- | --- | --- | --- | --- | --- | --- | --- | --- |
| Sex | female | 16 | 59.3 | 59.3 | 18 | 50.0 | 50.0 | 16 | 34.0 | 34.0 | 69 | 48.9 | 48.9 |
|  | male | 11 | 40.7 | 100.0 | 18 | 50.0 | 100.0 | 31 | 66.0 | 100.0 | 72 | 51.1 | 100.0 |
| p = 0.089 | all | 27 | 100.0 |  | 36 | 100.0 |  | 47 | 100.0 |  | 141 | 100.0 |  |
| Age | <45 | 21 | 77.8 | 77.8 | 25 | 69.4 | 69.4 | 39 | 83.0 | 83.0 | 110 | 78.0 | 78.0 |
|  | ≥45 | 6 | 22.2 | 100.0 | 11 | 30.6 | 100.0 | 8 | 17.0 | 100.0 | 31 | 22.0 | 100.0 |
| p = 0.344 | all | 27 | 100.0 |  | 36 | 100.0 |  | 47 | 100.0 |  | 141 | 100.0 |  |
| Histology | EN | 12 | 44.4 | 44.4 | 23 | 63.9 | 63.9 | 26 | 55.3 | 55.3 | 83 | 58.9 | 58.9 |
|  | other | 15 | 55.6 | 100.0 | 13 | 36.1 | 100.0 | 21 | 44.7 | 100.0 | 58 | 41.1 | 100.0 |
| p = 0.307 | all | 27 | 100.0 |  | 36 | 100.0 |  | 47 | 100.0 |  | 141 | 100.0 |  |
| B symptoms | No | 16 | 59.3 | 59.3 | 21 | 58.3 | 58.3 | 24 | 52.2 | 52.2 | 81 | 57.9 | 57.9 |
|  | Yes | 11 | 40.7 | 100.0 | 15 | 41.7 | 100.0 | 22 | 47.8 | 100.0 | 59 | 42.1 | 100.0 |
| p = 0.791 | all | 27 | 100.0 |  | 36 | 100.0 |  | 46 | 100.0 |  | 140 | 100.0 |  |
| Bulky mass | No | 21 | 77.8 | 77.8 | 31 | 86.1 | 86.1 | 40 | 85.1 | 85.1 | 112 | 79.4 | 79.4 |
|  | Yes | 6 | 22.2 | 100.0 | 5 | 13.9 | 100.0 | 7 | 14.9 | 100.0 | 29 | 20.6 | 100.0 |
| p = 0.634 | all | 27 | 100.0 |  | 36 | 100.0 |  | 47 | 100.0 |  | 141 | 100.0 |  |
| Anemia, Hb levels less than 10^5^ g/L | No | 20 | 74.1 | 74.1 | 30 | 83.3 | 83.3 | 34 | 72.3 | 72.3 | 110 | 78.0 | 78.0 |
|  | Yes | 7 | 25.9 | 100.0 | 6 | 16.7 | 100.0 | 13 | 27.7 | 100.0 | 31 | 22.0 | 100.0 |
| p = 0.480 | all | 27 | 100.0 |  | 36 | 100.0 |  | 47 | 100.0 |  | 141 | 100.0 |  |
| Leukocytosis, more than 15X10^9^/L | No | 23 | 85.2 | 85.2 | 33 | 94.3 | 94.3 | 38 | 80.8 | 80.8 | 123 | 87.9 | 87.9 |
|  | Yes | 4 | 14.8 | 100.0 | 2 | 5.7 | 100.0 | 9 | 19.1 | 100.0 | 17 | 12.1 | 100.0 |
| p = 0.214 | all | 27 | 100.0 |  | 35 | 100.0 |  | 47 | 100.0 |  | 140 | 100.0 |  |
| Lymphocytopenia, <0.6 X 10^9^/L or <8% of WBC | No | 25 | 92.6 | 92.6 | 29 | 82.9 | 82.9 | 39 | 86.7 | 86.7 | 121 | 87.7 | 87.7 |
|  | Yes | 2 | 7.4 | 100.0 | 6 | 17.1 | 100.0 | 6 | 13.3 | 100.0 | 17 | 12.3 | 100.0 |
| p = 0.529 | all | 27 | 100.0 |  | 35 | 100.0 |  | 45 | 100.0 |  | 138 | 100.0 |  |
| Hypoalbuminemia, <40 g/L | No | 15 | 62.5 | 62.5 | 27 | 79.4 | 79.4 | 30 | 68.2 | 68.2 | 86 | 65.2 | 65.2 |
|  | Yes | 9 | 37.5 | 100.0 | 7 | 20.6 | 100.0 | 14 | 31.8 | 100.0 | 46 | 34.9 | 100.0 |
| p = 0.341 | all | 24 | 100.0 |  | 34 | 100.0 |  | 44 | 100.0 |  | 132 | 100.0 |  |
| High LDH level, >450 UI/L | No | 20 | 74.1 | 74.1 | 25 | 71.4 | 71.4 | 31 | 67.4 | 67.4 | 99 | 71.2 | 71.2 |
|  | Yes | 7 | 25.9 | 100.0 | 10 | 28.6 | 100.0 | 15 | 32.6 | 100.0 | 40 | 28.8 | 100.0 |
| p = 0.822 | all | 27 | 100.0 |  | 35 | 100.0 |  | 46 | 100.0 |  | 139 | 100.0 |  |
| High B-2-microglobulin level, >25 mg/L | No | 14 | 70.0 | 70.0 | 22 | 78.6 | 78.6 | 27 | 71.0 | 71.0 | 84 | 75.7 | 75.7 |
|  | Yes | 6 | 30.0 | 100.0 | 6 | 21.4 | 100.0 | 11 | 28.9 | 100.0 | 27 | 24.3 | 100.0 |
| p = 0.738 | all | 20 | 100.0 |  | 28 | 100.0 |  | 38 | 100.0 |  | 111 | 100.0 |  |
| Stage | Early(I‐II) | 17 | 65.4 | 65.4 | 20 | 55.6 | 55.6 | 27 | 57.5 | 57.5 | 86 | 61.4 | 61.4 |
|  | Advanced (III‐IV) | 9 | 34.6 | 100.0 | 16 | 44.4 | 100.0 | 20 | 42.5 | 100.0 | 54 | 38.6 | 100.0 |
| p = 0.720 | all | 26 | 100.0 |  | 36 | 100.0 |  | 47 | 100.0 |  | 140 | 100.0 |  |
| Treatment | ABVD | 18 | 66.7 | 66.7 | 23 | 65.7 | 65.7 | 28 | 62.2 | 62.2 | 73 | 52.9 | 52.9 |
|  | MOPABV | 6 | 22.2 | 88.9 | 11 | 31.4 | 97.1 | 11 | 24.4 | 86.7 | 53 | 38.4 | 91.3 |
|  | MOPP | 3 | 11.1 | 100.0 | 0 | 0.0 | 97.1 | 3 | 6.7 | 93.3 | 8 | 5.8 | 97.1 |
|  | Other | 0 | 0.0 | 100.0 | 1 | 2.9 | 100.0 | 3 | 6.7 | 100.0 | 4 | 2.9 | 100.0 |
| p = 0.393 | all | 27 | 100.0 |  | 35 | 100.0 |  | 45 | 100.0 |  | 138 | 100.0 |  |
| EBV | No | 18 | 72.0 | 72.0 | 19 | 65.5 | 65.5 | 22 | 53.7 | 53.7 | 65 | 61.9 | 61.9 |
|  | Yes | 7 | 28.0 | 100.0 | 10 | 34.5 | 100.0 | 19 | 46.3 | 100.0 | 40 | 38.1 | 100.0 |
| p = 0.297 | all | 25 | 100.0 |  | 29 | 100.0 |  | 41 | 100.0 |  | 105 | 100.0 |  |
| Neutropenia toxicity | No | 18 | 66.7 | 66.7 | 24 | 66.7 | 66.7 | 28 | 59.6 | 59.6 | 94 | 66.7 | 66.7 |
|  | Yes | 9 | 33.3 | 100.0 | 12 | 33.3 | 100.0 | 19 | 40.4 | 100.0 | 47 | 33.3 | 100.0 |
| p = 0.746 | all | 27 | 100.0 |  | 36 | 100.0 |  | 47 | 100.0 |  | 141 | 100.0 |  |
| Anemia toxicity | No | 25 | 92.6 | 92.6 | 35 | 97.2 | 97.2 | 45 | 95.7 | 95.7 | 135 | 95.7 | 95.7 |
|  | Yes | 2 | 7.4 | 100.0 | 1 | 2.8 | 100.0 | 2 | 4.3 | 100.0 | 6 | 4.3 | 100.0 |
| p = 0.677 | all | 27 | 100.0 |  | 36 | 100.0 |  | 47 | 100.0 |  | 141 | 100.0 |  |
| Thrombocytopenia toxicity | No | 26 | 96.3 | 96.3 | 34 | 94.4 | 94.4 | 46 | 97.9 | 97.9 | 135 | 95.7 | 95.7 |
|  | Yes | 1 | 3.7 | 100.0 | 2 | 5.6 | 100.0 | 1 | 2.1 | 100.0 | 6 | 4.3 | 100.0 |
| p = 0.710 | all | 27 | 100.0 |  | 36 | 100.0 |  | 47 | 100.0 |  | 141 | 100.0 |  |
| Pulmonary toxicity | No | 23 | 88.5 | 88.5 | 36 | 100.0 | 100.0 | 45 | 95.7 | 95.7 | 133 | 95.0 | 95.0 |
|  | Yes | 3 | 11.5 | 100.0 | 0 | 0.0 | 100.0 | 2 | 4.3 | 100.0 | 7 | 5.0 | 100.0 |
| p = 0.100 | all | 26 | 100.0 |  | 36 | 100.0 |  | 47 | 100.0 |  | 140 | 100.0 |  |
| Neurological toxicity | No | 25 | 92.6 | 92.6 | 30 | 83.3 | 83.3 | 42 | 89.4 | 89.4 | 118 | 83.7 | 83.7 |
|  | Yes | 2 | 7.4 | 100.0 | 6 | 16.7 | 100.0 | 5 | 10.6 | 100.0 | 23 | 16.3 | 100.0 |
| p = 0.502 | all | 27 | 100.0 |  | 36 | 100.0 |  | 47 | 100.0 |  | 141 | 100.0 |  |
| Infectious toxicity | No | 17 | 63.0 | 63.0 | 26 | 72.2 | 72.2 | 28 | 59.6 | 59.6 | 90 | 63.8 | 63.8 |
|  | Yes | 10 | 37.0 | 100.0 | 10 | 27.8 | 100.0 | 19 | 40.4 | 100.0 | 51 | 36.2 | 100.0 |
| p = 0.481 | all | 27 | 100.0 |  | 36 | 100.0 |  | 47 | 100.0 |  | 141 | 100.0 |  |

1. **MIR146A rs2910164 (1🡪GG; 2🡪CC; 3🡪CG).**

| **Variable** | **Levels** | **N_1_** | **%_1_** | **∑%_1_** | **N_2_** | **%_2_** | **∑%_2_** | **N_3_** | **%_3_** | **∑%_3_** | **N_all_** | **%_all_** | **∑%_all_** |
| --- | --- | --- | --- | --- | --- | --- | --- | --- | --- | --- | --- | --- | --- |
| Sex | female | 35 | 48.0 | 48.0 | 4 | 44.4 | 44.4 | 30 | 50.9 | 50.9 | 69 | 48.9 | 48.9 |
|  | male | 38 | 52.0 | 100.0 | 5 | 55.6 | 100.0 | 29 | 49.1 | 100.0 | 72 | 51.1 | 100.0 |
| p = 0.910 | all | 73 | 100.0 |  | 9 | 100.0 |  | 59 | 100.0 |  | 141 | 100.0 |  |
| Age | <45 | 56 | 76.7 | 76.7 | 6 | 66.7 | 66.7 | 48 | 81.4 | 81.4 | 110 | 78.0 | 78.0 |
|  | ≥45 | 17 | 23.3 | 100.0 | 3 | 33.3 | 100.0 | 11 | 18.6 | 100.0 | 31 | 22.0 | 100.0 |
| p = 0.568 | all | 73 | 100.0 |  | 9 | 100.0 |  | 59 | 100.0 |  | 141 | 100.0 |  |
| Histology | EN | 46 | 63.0 | 63.0 | 4 | 44.4 | 44.4 | 33 | 55.9 | 55.9 | 83 | 58.9 | 58.9 |
|  | other | 27 | 37.0 | 100.0 | 5 | 55.6 | 100.0 | 26 | 44.1 | 100.0 | 58 | 41.1 | 100.0 |
| p = 0.472 | all | 73 | 100.0 |  | 9 | 100.0 |  | 59 | 100.0 |  | 141 | 100.0 |  |
| B symptoms | No | 44 | 60.3 | 60.3 | 3 | 33.3 | 33.3 | 34 | 58.6 | 58.6 | 81 | 57.9 | 57.9 |
|  | Yes | 29 | 39.7 | 100.0 | 6 | 66.7 | 100.0 | 24 | 41.4 | 100.0 | 59 | 42.1 | 100.0 |
| p = 0.300 | all | 73 | 100.0 |  | 9 | 100.0 |  | 58 | 100.0 |  | 140 | 100.0 |  |
| Bulky mass | No | 62 | 84.9 | 84.9 | 8 | 88.9 | 88.9 | 42 | 71.2 | 71.2 | 112 | 79.4 | 79.4 |
|  | Yes | 11 | 15.1 | 100.0 | 1 | 11.1 | 100.0 | 17 | 28.8 | 100.0 | 29 | 20.6 | 100.0 |
| p = 0.116 | all | 73 | 100.0 |  | 9 | 100.0 |  | 59 | 100.0 |  | 141 | 100.0 |  |
| Anemia, Hb levels less than 10^5^ g/L | No | 59 | 80.8 | 80.8 | 5 | 55.6 | 55.6 | 46 | 78.0 | 78.0 | 110 | 78.0 | 78.0 |
|  | Yes | 14 | 19.2 | 100.0 | 4 | 44.4 | 100.0 | 13 | 22.0 | 100.0 | 31 | 22.0 | 100.0 |
| p = 0.225 | all | 73 | 100.0 |  | 9 | 100.0 |  | 59 | 100.0 |  | 141 | 100.0 |  |
| Leukocytosis, more than 15X10^9^/L | No | 64 | 88.9 | 88.9 | 7 | 77.8 | 77.8 | 52 | 88.1 | 88.1 | 123 | 87.9 | 87.9 |
|  | Yes | 8 | 11.1 | 100.0 | 2 | 22.2 | 100.0 | 7 | 11.9 | 100.0 | 17 | 12.1 | 100.0 |
| p = 0.627 | all | 72 | 100.0 |  | 9 | 100.0 |  | 59 | 100.0 |  | 140 | 100.0 |  |
| Lymphocytopenia, <0.6 X 10^9^/L or <8% of WBC | No | 63 | 90.0 | 90.0 | 9 | 100.0 | 100.0 | 49 | 83.0 | 83.0 | 121 | 87.7 | 87.7 |
|  | Yes | 7 | 10.0 | 100.0 | 0 | 0.0 | 100.0 | 10 | 16.9 | 100.0 | 17 | 12.3 | 100.0 |
| p = 0.248 | all | 70 | 100.0 |  | 9 | 100.0 |  | 59 | 100.0 |  | 138 | 100.0 |  |
| Hypoalbuminemia, <40 g/L | No | 43 | 63.2 | 63.2 | 5 | 55.6 | 55.6 | 38 | 69.1 | 69.1 | 86 | 65.2 | 65.2 |
|  | Yes | 25 | 36.8 | 100.0 | 4 | 44.4 | 100.0 | 17 | 30.9 | 100.0 | 46 | 34.9 | 100.0 |
| p = 0.653 | all | 68 | 100.0 |  | 9 | 100.0 |  | 55 | 100.0 |  | 132 | 100.0 |  |
| High LDH level, >450 UI/L | No | 49 | 69.0 | 69.0 | 4 | 44.4 | 44.4 | 46 | 78.0 | 78.0 | 99 | 71.2 | 71.2 |
|  | Yes | 22 | 31.0 | 100.0 | 5 | 55.6 | 100.0 | 13 | 22.0 | 100.0 | 40 | 28.8 | 100.0 |
| p = 0.100 | all | 71 | 100.0 |  | 9 | 100.0 |  | 59 | 100.0 |  | 139 | 100.0 |  |
| High B-2-microglobulin level, >25 mg/L | No | 45 | 75.0 | 75.0 | 7 | 87.5 | 87.5 | 32 | 74.4 | 74.4 | 84 | 75.7 | 75.7 |
|  | Yes | 15 | 25.0 | 100.0 | 1 | 12.5 | 100.0 | 11 | 25.6 | 100.0 | 27 | 24.3 | 100.0 |
| p = 0.719 | all | 60 | 100.0 |  | 8 | 100.0 |  | 43 | 100.0 |  | 111 | 100.0 |  |
| Stage | Early(I‐II) | 40 | 55.6 | 55.6 | 5 | 55.6 | 55.6 | 41 | 69.5 | 69.5 | 86 | 61.4 | 61.4 |
|  | Advanced (III‐IV) | 32 | 44.4 | 100.0 | 4 | 44.4 | 100.0 | 18 | 30.5 | 100.0 | 54 | 38.6 | 100.0 |
| p = 0.247 | all | 72 | 100.0 |  | 9 | 100.0 |  | 59 | 100.0 |  | 140 | 100.0 |  |
| Treatment | ABVD | 39 | 54.2 | 54.2 | 4 | 44.4 | 44.4 | 30 | 52.6 | 52.6 | 73 | 52.9 | 52.9 |
|  | MOPABV | 29 | 40.3 | 94.5 | 2 | 22.2 | 66.7 | 22 | 38.6 | 91.2 | 53 | 38.4 | 91.3 |
|  | MOPP | 1 | 1.4 | 95.8 | 3 | 33.3 | 100.0 | 4 | 7.0 | 98.2 | 8 | 5.8 | 97.1 |
|  | Other | 3 | 4.2 | 100.0 | 0 | 0.0 | 100.0 | 1 | 1.8 | 100.0 | 4 | 2.9 | 100.0 |
| p = 0.013 | all | 72 | 100.0 |  | 9 | 100.0 |  | 57 | 100.0 |  | 138 | 100.0 |  |
| EBV | No | 37 | 67.3 | 67.3 | 3 | 42.9 | 42.9 | 25 | 58.1 | 58.1 | 65 | 61.9 | 61.9 |
|  | Yes | 18 | 32.7 | 100.0 | 4 | 57.1 | 100.0 | 18 | 41.9 | 100.0 | 40 | 38.1 | 100.0 |
| p = 0.366 | all | 55 | 100.0 |  | 7 | 100.0 |  | 43 | 100.0 |  | 105 | 100.0 |  |
| Neutropenia toxicity | No | 48 | 65.8 | 65.8 | 4 | 44.4 | 44.4 | 42 | 71.2 | 71.2 | 94 | 66.7 | 66.7 |
|  | Yes | 25 | 34.2 | 100.0 | 5 | 55.6 | 100.0 | 17 | 28.8 | 100.0 | 47 | 33.3 | 100.0 |
| p = 0.277 | all | 73 | 100.0 |  | 9 | 100.0 |  | 59 | 100.0 |  | 141 | 100.0 |  |
| Anemia toxicity | No | 70 | 95.9 | 95.9 | 9 | 100.0 | 100.0 | 56 | 94.9 | 94.9 | 135 | 95.7 | 95.7 |
|  | Yes | 3 | 4.1 | 100.0 | 0 | 0.0 | 100.0 | 3 | 5.1 | 100.0 | 6 | 4.3 | 100.0 |
| p =0.777 | all | 73 | 100.0 |  | 9 | 100.0 |  | 59 | 100.0 |  | 141 | 100.0 |  |
| Thrombocytopenia toxicity | No | 71 | 97.3 | 97.3 | 8 | 88.9 | 88.9 | 56 | 94.9 | 94.9 | 135 | 95.7 | 95.7 |
|  | Yes | 2 | 2.7 | 100.0 | 1 | 11.1 | 100.0 | 3 | 5.1 | 100.0 | 6 | 4.3 | 100.0 |
| p = 0.461 | all | 73 | 100.0 |  | 9 | 100.0 |  | 59 | 100.0 |  | 141 | 100.0 |  |
| Pulmonary toxicity | No | 69 | 94.5 | 94.5 | 8 | 100.0 | 100.0 | 56 | 94.9 | 94.9 | 133 | 95.0 | 95.0 |
|  | Yes | 4 | 5.5 | 100.0 | 0 | 0.0 | 100.0 | 3 | 5.1 | 100.0 | 7 | 5.0 | 100.0 |
| p = 0.796 | all | 73 | 100.0 |  | 8 | 100.0 |  | 59 | 100.0 |  | 140 | 100.0 |  |
| Neurological toxicity | No | 57 | 78.1 | 78.1 | 7 | 77.8 | 77.8 | 54 | 91.5 | 91.5 | 118 | 83.7 | 83.7 |
|  | Yes | 16 | 21.9 | 100.0 | 2 | 22.2 | 100.0 | 5 | 8.5 | 100.0 | 23 | 16.3 | 100.0 |
| p = 0.102 | all | 73 | 100.0 |  | 9 | 100.0 |  | 59 | 100.0 |  | 141 | 100.0 |  |
| Infectious toxicity | No | 45 | 61.6 | 61.6 | 5 | 55.6 | 55.6 | 40 | 67.8 | 67.8 | 90 | 63.8 | 63.8 |
|  | Yes | 28 | 38.4 | 100.0 | 4 | 44.4 | 100.0 | 19 | 32.2 | 100.0 | 51 | 36.2 | 100.0 |
| p = 0.664 | all | 73 | 100.0 |  | 9 | 100.0 |  | 59 | 100.0 |  | 141 | 100.0 |  |

1. **KRT81 rs3660 (1**🡪**GG; 2**🡪**CC; 3**🡪**CG).**

| **Variable** | **Levels** | **N_1_** | **%_1_** | **∑%_1_** | **N_2_** | **%_2_** | **∑%_2_** | **N_3_** | **%_3_** | **∑%_3_** | **N_all_** | **%_all_** | **∑%_all_** |
| --- | --- | --- | --- | --- | --- | --- | --- | --- | --- | --- | --- | --- | --- |
| Sex | female | 21 | 48.8 | 48.8 | 15 | 46.9 | 46.9 | 31 | 48.4 | 48.4 | 69 | 48.9 | 48.9 |
|  | male | 22 | 51.2 | 100.0 | 17 | 53.1 | 100.0 | 33 | 51.6 | 100.0 | 72 | 51.1 | 100.0 |
| p = 0.985 | all | 43 | 100.0 |  | 32 | 100.0 |  | 64 | 100.0 |  | 141 | 100.0 |  |
| Age | <45 | 32 | 74.4 | 74.4 | 26 | 81.2 | 81.2 | 51 | 79.7 | 79.7 | 110 | 78.0 | 78.0 |
|  | ≥45 | 11 | 25.6 | 100.0 | 6 | 18.8 | 100.0 | 13 | 20.3 | 100.0 | 31 | 22.0 | 100.0 |
| p = 0.734 | all | 43 | 100.0 |  | 32 | 100.0 |  | 64 | 100.0 |  | 141 | 100.0 |  |
| Histology | EN | 25 | 58.1 | 58.1 | 19 | 59.4 | 59.4 | 37 | 57.8 | 57.8 | 83 | 58.9 | 58.9 |
|  | other | 18 | 41.9 | 100.0 | 13 | 40.6 | 100.0 | 27 | 42.2 | 100.0 | 58 | 41.1 | 100.0 |
| p = 0.989 | all | 43 | 100.0 |  | 32 | 100.0 |  | 64 | 100.0 |  | 141 | 100.0 |  |
| B symptoms | No | 25 | 58.1 | 58.1 | 18 | 56.2 | 56.2 | 36 | 57.1 | 57.1 | 81 | 57.9 | 57.9 |
|  | Yes | 18 | 41.9 | 100.0 | 14 | 43.8 | 100.0 | 27 | 42.9 | 100.0 | 59 | 42.1 | 100.0 |
| p = 0.986 | all | 43 | 100.0 |  | 32 | 100.0 |  | 63 | 100.0 |  | 140 | 100.0 |  |
| Bulky mass | No | 30 | 69.8 | 69.8 | 26 | 81.2 | 81.2 | 55 | 85.9 | 85.9 | 112 | 79.4 | 79.4 |
|  | Yes | 13 | 30.2 | 100.0 | 6 | 18.8 | 100.0 | 9 | 14.1 | 100.0 | 29 | 20.6 | 100.0 |
| p = 0.120 | all | 43 | 100.0 |  | 32 | 100.0 |  | 64 | 100.0 |  | 141 | 100.0 |  |
| Anemia, Hb levels less than 10^5^ g/L | No | 33 | 76.7 | 76.7 | 25 | 78.1 | 78.1 | 50 | 78.1 | 78.1 | 110 | 78.0 | 78.0 |
|  | Yes | 10 | 23.3 | 100.0 | 7 | 21.9 | 100.0 | 14 | 21.9 | 100.0 | 31 | 22.0 | 100.0 |
| p = 0.984 | all | 43 | 100.0 |  | 32 | 100.0 |  | 64 | 100.0 |  | 141 | 100.0 |  |
| Leukocytosis, more than 15X10^9^/L | No | 37 | 86.0 | 86.0 | 29 | 90.6 | 90.6 | 56 | 88.9 | 88.9 | 123 | 87.9 | 87.9 |
|  | Yes | 6 | 13.9 | 100.0 | 3 | 9.4 | 100.0 | 7 | 11.1 | 100.0 | 17 | 12.1 | 100.0 |
| p = 0.818 | all | 43 | 100.0 |  | 32 | 100.0 |  | 63 | 100.0 |  | 140 | 100.0 |  |
| Lymphocytopenia, <0.6 X 10^9^/L or <8% of WBC | No | 36 | 85.7 | 85.7 | 29 | 90.6 | 90.6 | 54 | 87.1 | 87.1 | 121 | 87.7 | 87.7 |
|  | Yes | 6 | 14.3 | 100.0 | 3 | 9.4 | 100.0 | 8 | 12.9 | 100.0 | 17 | 12.3 | 100.0 |
| p = 0.812 | all | 42 | 100.0 |  | 32 | 100.0 |  | 62 | 100.0 |  | 138 | 100.0 |  |
| Hypoalbuminemia, <40 g/L | No | 26 | 68.4 | 68.4 | 22 | 71.0 | 71.0 | 37 | 60.7 | 60.7 | 86 | 65.2 | 65.2 |
|  | Yes | 12 | 31.6 | 100.0 | 9 | 29.0 | 100.0 | 24 | 39.3 | 100.0 | 46 | 34.9 | 100.0 |
| p = 0.553 | all | 38 | 100.0 |  | 31 | 100.0 |  | 61 | 100.0 |  | 132 | 100.0 |  |
| High LDH level, >450 UI/L | No | 29 | 69.0 | 69.0 | 20 | 64.5 | 64.5 | 48 | 75.0 | 75.0 | 99 | 71.2 | 71.2 |
|  | Yes | 13 | 30.9 | 100.0 | 11 | 35.5 | 100.0 | 16 | 25.0 | 100.0 | 40 | 28.8 | 100.0 |
| p = 0.548 | all | 42 | 100.0 |  | 31 | 100.0 |  | 64 | 100.0 |  | 139 | 100.0 |  |
| High B-2-microglobulin level, >25 mg/L | No | 24 | 75.0 | 75.0 | 18 | 72.0 | 72.0 | 41 | 77.4 | 77.4 | 84 | 75.7 | 75.7 |
|  | Yes | 8 | 25.0 | 100.0 | 7 | 28.0 | 100.0 | 12 | 22.6 | 100.0 | 27 | 24.3 | 100.0 |
| p = 0.874 | all | 32 | 100.0 |  | 25 | 100.0 |  | 53 | 100.0 |  | 111 | 100.0 |  |
| Stage | Early(I‐II) | 27 | 62.8 | 62.8 | 23 | 71.9 | 71.9 | 35 | 55.6 | 55.6 | 86 | 61.4 | 61.4 |
|  | Advanced (III‐IV) | 16 | 37.2 | 100.0 | 9 | 28.1 | 100.0 | 28 | 44.4 | 100.0 | 54 | 38.6 | 100.0 |
| p = 0.297 | all | 43 | 100.0 |  | 32 | 100.0 |  | 63 | 100.0 |  | 140 | 100.0 |  |
| Treatment | ABVD | 23 | 56.1 | 56.1 | 16 | 50.0 | 50.0 | 34 | 54.0 | 54.0 | 73 | 52.9 | 52.9 |
|  | MOPABV | 13 | 31.7 | 87.8 | 13 | 40.6 | 90.6 | 25 | 39.7 | 93.7 | 53 | 38.4 | 91.3 |
|  | MOPP | 3 | 7.3 | 95.1 | 3 | 9.4 | 100.0 | 2 | 3.2 | 96.8 | 8 | 5.8 | 97.1 |
|  | Other | 2 | 4.9 | 100.0 | 0 | 0.0 | 100.0 | 2 | 3.2 | 100.0 | 4 | 2.9 | 100.0 |
| p = 0.714 | all | 41 | 100.0 |  | 32 | 100.0 |  | 63 | 100.0 |  | 138 | 100.0 |  |
| EBV | No | 21 | 65.6 | 65.6 | 13 | 59.1 | 59.1 | 30 | 60.0 | 60.0 | 65 | 61.9 | 61.9 |
|  | Yes | 11 | 34.4 | 100.0 | 9 | 40.9 | 100.0 | 20 | 40.0 | 100.0 | 40 | 38.1 | 100.0 |
| p = 0.847 | all | 32 | 100.0 |  | 22 | 100.0 |  | 50 | 100.0 |  | 105 | 100.0 |  |
| Neutropenia toxicity | No | 28 | 65.1 | 65.1 | 19 | 59.4 | 59.4 | 45 | 70.3 | 70.3 | 94 | 66.7 | 66.7 |
|  | Yes | 15 | 34.9 | 100.0 | 13 | 40.6 | 100.0 | 19 | 29.7 | 100.0 | 47 | 33.3 | 100.0 |
| p = 0.556 | all | 43 | 100.0 |  | 32 | 100.0 |  | 64 | 100.0 |  | 141 | 100.0 |  |
| Anemia toxicity | No | 40 | 93.0 | 93.0 | 31 | 96.9 | 96.9 | 62 | 96.9 | 96.9 | 135 | 95.7 | 95.7 |
|  | Yes | 3 | 7.0 | 100.0 | 1 | 3.1 | 100.0 | 2 | 3.1 | 100.0 | 6 | 4.3 | 100.0 |
| p = 0.586 | all | 43 | 100.0 |  | 32 | 100.0 |  | 64 | 100.0 |  | 141 | 100.0 |  |
| Thrombocytopenia toxicity | No | 40 | 93.0 | 93.0 | 32 | 100.0 | 100.0 | 61 | 95.3 | 95.3 | 135 | 95.7 | 95.7 |
|  | Yes | 3 | 7.0 | 100.0 | 0 | 0.0 | 100.0 | 3 | 4.7 | 100.0 | 6 | 4.3 | 100.0 |
| p = 0.332 | all | 43 | 100.0 |  | 32 | 100.0 |  | 64 | 100.0 |  | 141 | 100.0 |  |
| Pulmonary toxicity | No | 41 | 97.6 | 97.6 | 31 | 96.9 | 96.9 | 59 | 92.2 | 92.2 | 133 | 95.0 | 95.0 |
|  | Yes | 1 | 2.4 | 100.0 | 1 | 3.1 | 100.0 | 5 | 7.8 | 100.0 | 7 | 5.0 | 100.0 |
| p = 0.390 | all | 42 | 100.0 |  | 32 | 100.0 |  | 64 | 100.0 |  | 140 | 100.0 |  |
| Neurological toxicity | No | 38 | 88.4 | 88.4 | 22 | 68.8 | 68.8 | 56 | 87.5 | 87.5 | 118 | 83.7 | 83.7 |
|  | Yes | 5 | 11.6 | 100.0 | 10 | 31.2 | 100.0 | 8 | 12.5 | 100.0 | 23 | 16.3 | 100.0 |
| p = 0.038 | all | 43 | 100.0 |  | 32 | 100.0 |  | 64 | 100.0 |  | 141 | 100.0 |  |
| Infectious toxicity | No | 28 | 65.1 | 65.1 | 18 | 56.2 | 56.2 | 42 | 65.6 | 65.6 | 90 | 63.8 | 63.8 |
|  | Yes | 15 | 34.9 | 100.0 | 14 | 43.8 | 100.0 | 22 | 34.4 | 100.0 | 51 | 36.2 | 100.0 |
| p = 0.639 | all | 43 | 100.0 |  | 32 | 100.0 |  | 64 | 100.0 |  | 141 | 100.0 |  |

1. **FAM179B rs1053667 (1**🡪**TT; 2**🡪**CC; 3**🡪**CT).**

| **Variable** | **Levels** | **N_1_** | **%_1_** | **∑%_1_** | **N_2_** | **%_2_** | **∑%_2_** | **N_3_** | **%_3_** | **∑%_3_** | **N_all_** | **%_all_** | **∑%_all_** |
| --- | --- | --- | --- | --- | --- | --- | --- | --- | --- | --- | --- | --- | --- |
| Sex | female | 44 | 46.3 | 46.3 | 1 | 100.0 | 100.0 | 2 | 28.6 | 28.6 | 69 | 48.9 | 48.9 |
|  | male | 51 | 53.7 | 100.0 | 0 | 0.0 | 100.0 | 5 | 71.4 | 100.0 | 72 | 51.1 | 100.0 |
| p = 0.362 | all | 95 | 100.0 |  | 1 | 100.0 |  | 7 | 100.0 |  | 141 | 100.0 |  |
| Age | <45 | 78 | 82.1 | 82.1 | 0 | 0.0 | 0.0 | 6 | 85.7 | 85.7 | 110 | 78.0 | 78.0 |
|  | ≥45 | 17 | 17.9 | 100.0 | 1 | 100.0 | 100.0 | 1 | 14.3 | 100.0 | 31 | 22.0 | 100.0 |
| p = 0.104 | all | 95 | 100.0 |  | 1 | 100.0 |  | 7 | 100.0 |  | 141 | 100.0 |  |
| Histology | EN | 55 | 57.9 | 57.9 | 0 | 0.0 | 0.0 | 4 | 57.1 | 57.1 | 83 | 58.9 | 58.9 |
|  | other | 40 | 42.1 | 100.0 | 1 | 100.0 | 100.0 | 3 | 42.9 | 100.0 | 58 | 41.1 | 100.0 |
| p = 0.508 | all | 95 | 100.0 |  | 1 | 100.0 |  | 7 | 100.0 |  | 141 | 100.0 |  |
| B symptoms | No | 56 | 59.6 | 59.6 | 0 | 0.0 | 0.0 | 6 | 85.7 | 85.7 | 81 | 57.9 | 57.9 |
|  | Yes | 38 | 40.4 | 100.0 | 1 | 100.0 | 100.0 | 1 | 14.3 | 100.0 | 59 | 42.1 | 100.0 |
| p = 0.180 | all | 94 | 100.0 |  | 1 | 100.0 |  | 7 | 100.0 |  | 140 | 100.0 |  |
| Bulky mass | No | 77 | 81.0 | 81.0 | 1 | 100.0 | 100.0 | 7 | 100.0 | 100.0 | 112 | 79.4 | 79.4 |
|  | Yes | 18 | 18.9 | 100.0 | 0 | 0.0 | 100.0 | 0 | 0.0 | 100.0 | 29 | 20.6 | 100.0 |
| p = 0.399 | all | 95 | 100.0 |  | 1 | 100.0 |  | 7 | 100.0 |  | 141 | 100.0 |  |
| Anemia, Hb levels less than 10^5^ g/L | No | 74 | 77.9 | 77.9 | 0 | 0.0 | 0.0 | 6 | 85.7 | 85.7 | 110 | 78.0 | 78.0 |
|  | Yes | 21 | 22.1 | 100.0 | 1 | 100.0 | 100.0 | 1 | 14.3 | 100.0 | 31 | 22.0 | 100.0 |
| p = 0.154 | all | 95 | 100.0 |  | 1 | 100.0 |  | 7 | 100.0 |  | 141 | 100.0 |  |
| Leukocytosis, more than 15X10^9^/L | No | 79 | 84.0 | 84.0 | 1 | 100.0 | 100.0 | 7 | 100.0 | 100.0 | 123 | 87.9 | 87.9 |
|  | Yes | 15 | 16.0 | 100.0 | 0 | 0.0 | 100.0 | 0 | 0.0 | 100.0 | 17 | 12.1 | 100.0 |
| p = 0.473 | all | 94 | 100.0 |  | 1 | 100.0 |  | 7 | 100.0 |  | 140 | 100.0 |  |
| Lymphocytopenia, <0.6 X 10^9^/L or <8% of WBC | No | 80 | 87.0 | 87.0 | 0 | 0.0 | 0.0 | 7 | 100.0 | 100.0 | 121 | 87.7 | 87.7 |
|  | Yes | 12 | 13.0 | 100.0 | 1 | 100.0 | 100.0 | 0 | 0.0 | 100.0 | 17 | 12.3 | 100.0 |
| p = 0.021 | all | 92 | 100.0 |  | 1 | 100.0 |  | 7 | 100.0 |  | 138 | 100.0 |  |
| Hypoalbuminemia, <40 g/L | No | 63 | 71.6 | 71.6 | 0 | 0.0 | 0.0 | 5 | 83.3 | 83.3 | 86 | 65.2 | 65.2 |
|  | Yes | 25 | 28.4 | 100.0 | 1 | 100.0 | 100.0 | 1 | 16.7 | 100.0 | 46 | 34.9 | 100.0 |
| p = 0.231 | all | 88 | 100.0 |  | 1 | 100.0 |  | 6 | 100.0 |  | 132 | 100.0 |  |
| High LDH level, >450 UI/L | No | 65 | 69.9 | 69.9 | 1 | 100.0 | 100.0 | 6 | 85.7 | 85.7 | 99 | 71.2 | 71.2 |
|  | Yes | 28 | 30.1 | 100.0 | 0 | 0.0 | 100.0 | 1 | 14.3 | 100.0 | 40 | 28.8 | 100.0 |
| p = 0.483 | all | 93 | 100.0 |  | 1 | 100.0 |  | 7 | 100.0 |  | 139 | 100.0 |  |
| High B-2-microglobulin level, >25 mg/L | No | 57 | 76.0 | 76.0 | 0 | 0.0 | 0.0 | 3 | 75.0 | 75.0 | 84 | 75.7 | 75.7 |
|  | Yes | 18 | 24.0 | 100.0 | 1 | 100.0 | 100.0 | 1 | 25.0 | 100.0 | 27 | 24.3 | 100.0 |
| p = 0.219 | all | 75 | 100.0 |  | 1 | 100.0 |  | 4 | 100.0 |  | 111 | 100.0 |  |
| Stage | Early(I‐II) | 57 | 60.6 | 60.6 | 0 | 0.0 | 0.0 | 5 | 71.4 | 71.4 | 86 | 61.4 | 61.4 |
|  | Advanced (III‐IV) | 37 | 39.4 | 100.0 | 1 | 100.0 | 100.0 | 2 | 28.6 | 100.0 | 54 | 38.6 | 100.0 |
| p = 0.390 | all | 94 | 100.0 |  | 1 | 100.0 |  | 7 | 100.0 |  | 140 | 100.0 |  |
| Treatment | ABVD | 61 | 65.6 | 65.6 | 1 | 100.0 | 100.0 | 5 | 71.4 | 71.4 | 73 | 52.9 | 52.9 |
|  | MOPABV | 26 | 28.0 | 93.6 | 0 | 0.0 | 100.0 | 0 | 0.0 | 71.4 | 53 | 38.4 | 91.3 |
|  | MOPP | 5 | 5.4 | 98.9 | 0 | 0.0 | 100.0 | 0 | 0.0 | 71.4 | 8 | 5.8 | 97.1 |
|  | Other | 1 | 1.1 | 100.0 | 0 | 0.0 | 100.0 | 2 | 28.6 | 100.0 | 4 | 2.9 | 100.0 |
| p = 0.003 | all | 93 | 100.0 |  | 1 | 100.0 |  | 7 | 100.0 |  | 138 | 100.0 |  |
| EBV | No | 50 | 63.3 | 63.3 | 0 | 0.0 | 0.0 | 5 | 71.4 | 71.4 | 65 | 61.9 | 61.9 |
|  | Yes | 29 | 36.7 | 100.0 | 1 | 100.0 | 100.0 | 2 | 28.6 | 100.0 | 40 | 38.1 | 100.0 |
| p = 0.382 | all | 79 | 100.0 |  | 1 | 100.0 |  | 7 | 100.0 |  | 105 | 100.0 |  |
| Neutropenia toxicity | No | 64 | 67.4 | 67.4 | 0 | 0.0 | 0.0 | 3 | 42.9 | 42.9 | 94 | 66.7 | 66.7 |
|  | Yes | 31 | 32.6 | 100.0 | 1 | 100.0 | 100.0 | 4 | 57.1 | 100.0 | 47 | 33.3 | 100.0 |
| p = 0.165 | all | 95 | 100.0 |  | 1 | 100.0 |  | 7 | 100.0 |  | 141 | 100.0 |  |
| Anemia toxicity | No | 92 | 96.8 | 96.8 | 1 | 100.0 | 100.0 | 6 | 85.7 | 85.7 | 135 | 95.7 | 95.7 |
|  | Yes | 3 | 3.2 | 100.0 | 0 | 0.0 | 100.0 | 1 | 14.3 | 100.0 | 6 | 4.3 | 100.0 |
| p = 0.332 | all | 95 | 100.0 |  | 1 | 100.0 |  | 7 | 100.0 |  | 141 | 100.0 |  |
| Thrombocytopenia toxicity | No | 93 | 97.9 | 97.9 | 1 | 100.0 | 100.0 | 7 | 100.0 | 100.0 | 135 | 95.7 | 95.7 |
|  | Yes | 2 | 2.1 | 100.0 | 0 | 0.0 | 100.0 | 0 | 0.0 | 100.0 | 6 | 4.3 | 100.0 |
| p = 0.917 | all | 95 | 100.0 |  | 1 | 100.0 |  | 7 | 100.0 |  | 141 | 100.0 |  |
| Pulmonary toxicity | No | 89 | 94.7 | 94.7 | 1 | 100.0 | 100.0 | 7 | 100.0 | 100.0 | 133 | 95.0 | 95.0 |
|  | Yes | 5 | 5.3 | 100.0 | 0 | 0.0 | 100.0 | 0 | 0.0 | 100.0 | 7 | 5.0 | 100.0 |
| p = 0.800 | all | 94 | 100.0 |  | 1 | 100.0 |  | 7 | 100.0 |  | 140 | 100.0 |  |
| Neurological toxicity | No | 83 | 87.4 | 87.4 | 1 | 100.0 | 100.0 | 7 | 100.0 | 100.0 | 118 | 83.7 | 83.7 |
|  | Yes | 12 | 12.6 | 100.0 | 0 | 0.0 | 100.0 | 0 | 0.0 | 100.0 | 23 | 16.3 | 100.0 |
| p = 0.564 | all | 95 | 100.0 |  | 1 | 100.0 |  | 7 | 100.0 |  | 141 | 100.0 |  |
| Infectious toxicity | No | 60 | 63.2 | 63.2 | 0 | 0.0 | 0.0 | 4 | 57.1 | 57.1 | 90 | 63.8 | 63.8 |
|  | Yes | 35 | 36.8 | 100.0 | 1 | 100.0 | 100.0 | 3 | 42.9 | 100.0 | 51 | 36.2 | 100.0 |
| p = 0.415 | all | 95 | 100.0 |  | 1 | 100.0 |  | 7 | 100.0 |  | 141 | 100.0 |  |

1. **XPO5 rs11077 (1**🡪**CC; 2**🡪**AA; 3**🡪**AC).**

| **Variable** | **Levels** | **N_1_** | **%_1_** | **∑%_1_** | **N_2_** | **%_2_** | **∑%_2_** | **N_3_** | **%_3_** | **∑%_3_** | **N_all_** | **%_all_** | **∑%_all_** |
| --- | --- | --- | --- | --- | --- | --- | --- | --- | --- | --- | --- | --- | --- |
| Sex | female | 19 | 54.3 | 54.3 | 12 | 48.0 | 48.0 | 30 | 44.8 | 44.8 | 69 | 48.9 | 48.9 |
|  | male | 16 | 45.7 | 100.0 | 13 | 52.0 | 100.0 | 37 | 55.2 | 100.0 | 72 | 51.1 | 100.0 |
| p = 0.659 | all | 35 | 100.0 |  | 25 | 100.0 |  | 67 | 100.0 |  | 141 | 100.0 |  |
| Age | <45 | 25 | 71.4 | 71.4 | 23 | 92.0 | 92.0 | 49 | 73.1 | 73.1 | 110 | 78.0 | 78.0 |
|  | ≥45 | 10 | 28.6 | 100.0 | 2 | 8.0 | 100.0 | 18 | 26.9 | 100.0 | 31 | 22.0 | 100.0 |
| p = 0.120 | all | 35 | 100.0 |  | 25 | 100.0 |  | 67 | 100.0 |  | 141 | 100.0 |  |
| Histology | EN | 17 | 48.6 | 48.6 | 15 | 60.0 | 60.0 | 39 | 58.2 | 58.2 | 83 | 58.9 | 58.9 |
|  | other | 18 | 51.4 | 100.0 | 10 | 40.0 | 100.0 | 28 | 41.8 | 100.0 | 58 | 41.1 | 100.0 |
| p = 0.583 | all | 35 | 100.0 |  | 25 | 100.0 |  | 67 | 100.0 |  | 141 | 100.0 |  |
| B symptoms | No | 18 | 51.4 | 51.4 | 12 | 48.0 | 48.0 | 41 | 62.1 | 62.1 | 81 | 57.9 | 57.9 |
|  | Yes | 17 | 48.6 | 100.0 | 13 | 52.0 | 100.0 | 25 | 37.9 | 100.0 | 59 | 42.1 | 100.0 |
| p = 0.378 | all | 35 | 100.0 |  | 25 | 100.0 |  | 66 | 100.0 |  | 140 | 100.0 |  |
| Bulky mass | No | 24 | 68.6 | 68.6 | 19 | 76.0 | 76.0 | 61 | 91.0 | 91.0 | 112 | 79.4 | 79.4 |
|  | Yes | 11 | 31.4 | 100.0 | 6 | 24.0 | 100.0 | 6 | 9.0 | 100.0 | 29 | 20.6 | 100.0 |
| p = 0.014 | all | 35 | 100.0 |  | 25 | 100.0 |  | 67 | 100.0 |  | 141 | 100.0 |  |
| Anemia, Hb levels less than 10^5^ g/L | No | 25 | 71.4 | 71.4 | 18 | 72.0 | 72.0 | 54 | 80.6 | 80.6 | 110 | 78.0 | 78.0 |
|  | Yes | 10 | 28.6 | 100.0 | 7 | 28.0 | 100.0 | 13 | 19.4 | 100.0 | 31 | 22.0 | 100.0 |
| p = 0.496 | all | 35 | 100.0 |  | 25 | 100.0 |  | 67 | 100.0 |  | 141 | 100.0 |  |
| Leukocytosis, more than 15X10^9^/L | No | 30 | 85.7 | 85.7 | 22 | 88.0 | 88.0 | 58 | 87.9 | 87.9 | 123 | 87.9 | 87.9 |
|  | Yes | 5 | 14.3 | 100.0 | 3 | 12.0 | 100.0 | 8 | 12.1 | 100.0 | 17 | 12.1 | 100.0 |
| p = 0.946 | all | 35 | 100.0 |  | 25 | 100.0 |  | 66 | 100.0 |  | 140 | 100.0 |  |
| Lymphocytopenia, <0.6 X 10^9^/L or <8% of WBC | No | 30 | 85.7 | 85.7 | 18 | 72.0 | 72.0 | 59 | 92.2 | 92.2 | 121 | 87.7 | 87.7 |
|  | Yes | 5 | 14.3 | 100.0 | 7 | 28.0 | 100.0 | 5 | 7.8 | 100.0 | 17 | 12.3 | 100.0 |
| p = 0.045 | all | 35 | 100.0 |  | 25 | 100.0 |  | 64 | 100.0 |  | 138 | 100.0 |  |
| Hypoalbuminemia, <40 g/L | No | 21 | 61.8 | 61.8 | 16 | 66.7 | 66.7 | 44 | 72.1 | 72.1 | 86 | 65.2 | 65.2 |
|  | Yes | 13 | 38.2 | 100.0 | 8 | 33.3 | 100.0 | 17 | 27.9 | 100.0 | 46 | 34.9 | 100.0 |
| p = 0.575 | all | 34 | 100.0 |  | 24 | 100.0 |  | 61 | 100.0 |  | 132 | 100.0 |  |
| High LDH level, >450 UI/L | No | 24 | 68.6 | 68.6 | 16 | 64.0 | 64.0 | 49 | 75.4 | 75.4 | 99 | 71.2 | 71.2 |
|  | Yes | 11 | 31.4 | 100.0 | 9 | 36.0 | 100.0 | 16 | 24.6 | 100.0 | 40 | 28.8 | 100.0 |
| p = 0.521 | all | 35 | 100.0 |  | 25 | 100.0 |  | 65 | 100.0 |  | 139 | 100.0 |  |
| High B-2-microglobulin level, >25 mg/L | No | 21 | 72.4 | 72.4 | 13 | 65.0 | 65.0 | 40 | 78.4 | 78.4 | 84 | 75.7 | 75.7 |
|  | Yes | 8 | 27.6 | 100.0 | 7 | 35.0 | 100.0 | 11 | 21.6 | 100.0 | 27 | 24.3 | 100.0 |
| p = 0.496 | all | 29 | 100.0 |  | 20 | 100.0 |  | 51 | 100.0 |  | 111 | 100.0 |  |
| Stage | Early(I‐II) | 20 | 57.1 | 57.1 | 11 | 45.8 | 45.8 | 44 | 65.7 | 65.7 | 86 | 61.4 | 61.4 |
|  | Advanced (III‐IV) | 15 | 42.9 | 100.0 | 13 | 54.2 | 100.0 | 23 | 34.3 | 100.0 | 54 | 38.6 | 100.0 |
| p = 0.223 | all | 35 | 100.0 |  | 24 | 100.0 |  | 67 | 100.0 |  | 140 | 100.0 |  |
| Treatment | ABVD | 18 | 52.9 | 52.9 | 12 | 48.0 | 48.0 | 42 | 64.6 | 64.6 | 73 | 52.9 | 52.9 |
|  | MOPABV | 12 | 35.3 | 88.2 | 13 | 52.0 | 100.0 | 16 | 24.6 | 89.2 | 53 | 38.4 | 91.3 |
|  | MOPP | 2 | 5.9 | 94.1 | 0 | 0.0 | 100.0 | 5 | 7.7 | 96.9 | 8 | 5.8 | 97.1 |
|  | Other | 2 | 5.9 | 100.0 | 0 | 0.0 | 100.0 | 2 | 3.1 | 100.0 | 4 | 2.9 | 100.0 |
| p = 0.192 | all | 34 | 100.0 |  | 25 | 100.0 |  | 65 | 100.0 |  | 138 | 100.0 |  |
| EBV | No | 14 | 56.0 | 56.0 | 13 | 56.5 | 56.5 | 36 | 66.7 | 66.7 | 65 | 61.9 | 61.9 |
|  | Yes | 11 | 44.0 | 100.0 | 10 | 43.5 | 100.0 | 18 | 33.3 | 100.0 | 40 | 38.1 | 100.0 |
| p = 0.557 | all | 25 | 100.0 |  | 23 | 100.0 |  | 54 | 100.0 |  | 105 | 100.0 |  |
| Neutropenia toxicity | No | 23 | 65.7 | 65.7 | 17 | 68.0 | 68.0 | 42 | 62.7 | 62.7 | 94 | 66.7 | 66.7 |
|  | Yes | 12 | 34.3 | 100.0 | 8 | 32.0 | 100.0 | 25 | 37.3 | 100.0 | 47 | 33.3 | 100.0 |
| p = 0.882 | all | 35 | 100.0 |  | 25 | 100.0 |  | 67 | 100.0 |  | 141 | 100.0 |  |
| Anemia toxicity | No | 32 | 91.4 | 91.4 | 25 | 100.0 | 100.0 | 64 | 95.5 | 95.5 | 135 | 95.7 | 95.7 |
|  | Yes | 3 | 8.6 | 100.0 | 0 | 0.0 | 100.0 | 3 | 4.5 | 100.0 | 6 | 4.3 | 100.0 |
| p = 0.301 | all | 35 | 100.0 |  | 25 | 100.0 |  | 67 | 100.0 |  | 141 | 100.0 |  |
| Thrombocytopenia toxicity | No | 32 | 91.4 | 91.4 | 25 | 100.0 | 100.0 | 65 | 97.0 | 97.0 | 135 | 95.7 | 95.7 |
|  | Yes | 3 | 8.6 | 100.0 | 0 | 0.0 | 100.0 | 2 | 3.0 | 100.0 | 6 | 4.3 | 100.0 |
| p = 0.205 | all | 35 | 100.0 |  | 25 | 100.0 |  | 67 | 100.0 |  | 141 | 100.0 |  |
| Pulmonary toxicity | No | 32 | 91.4 | 91.4 | 22 | 88.0 | 88.0 | 65 | 98.5 | 98.5 | 133 | 95.0 | 95.0 |
|  | Yes | 3 | 8.6 | 100.0 | 3 | 12.0 | 100.0 | 1 | 1.5 | 100.0 | 7 | 5.0 | 100.0 |
| p = 0.098 | all | 35 | 100.0 |  | 25 | 100.0 |  | 66 | 100.0 |  | 140 | 100.0 |  |
| Neurological toxicity | No | 31 | 88.6 | 88.6 | 19 | 76.0 | 76.0 | 61 | 91.0 | 91.0 | 118 | 83.7 | 83.7 |
|  | Yes | 4 | 11.4 | 100.0 | 6 | 24.0 | 100.0 | 6 | 9.0 | 100.0 | 23 | 16.3 | 100.0 |
| p = 0.149 | all | 35 | 100.0 |  | 25 | 100.0 |  | 67 | 100.0 |  | 141 | 100.0 |  |
| Infectious toxicity | No | 23 | 65.7 | 65.7 | 17 | 68.0 | 68.0 | 40 | 59.7 | 59.7 | 90 | 63.8 | 63.8 |
|  | Yes | 12 | 34.3 | 100.0 | 8 | 32.0 | 100.0 | 27 | 40.3 | 100.0 | 51 | 36.2 | 100.0 |
| p = 0.708 | all | 35 | 100.0 |  | 25 | 100.0 |  | 67 | 100.0 |  | 141 | 100.0 |  |

1. **TRBP rs784567 (1🡪CC; 2🡪TT; 3🡪CT).**

| **Variable** | **Levels** | **N_1_** | **%_1_** | **∑%_1_** | **N_2_** | **%_2_** | **∑%_2_** | **N_3_** | **%_3_** | **∑%_3_** | **N_all_** | **%_all_** | **∑%_all_** |
| --- | --- | --- | --- | --- | --- | --- | --- | --- | --- | --- | --- | --- | --- |
| Sex | female | 24 | 60.0 | 60.0 | 18 | 58.1 | 58.1 | 25 | 36.8 | 36.8 | 69 | 48.9 | 48.9 |
|  | male | 16 | 40.0 | 100.0 | 13 | 41.9 | 100.0 | 43 | 63.2 | 100.0 | 72 | 51.1 | 100.0 |
| p = 0.030 | all | 40 | 100.0 |  | 31 | 100.0 |  | 68 | 100.0 |  | 141 | 100.0 |  |
| Age | <45 | 35 | 87.5 | 87.5 | 23 | 74.2 | 74.2 | 51 | 75.0 | 75.0 | 110 | 78.0 | 78.0 |
|  | ≥45 | 5 | 12.5 | 100.0 | 8 | 25.8 | 100.0 | 17 | 25.0 | 100.0 | 31 | 22.0 | 100.0 |
| p = 0.253 | all | 40 | 100.0 |  | 31 | 100.0 |  | 68 | 100.0 |  | 141 | 100.0 |  |
| Histology | EN | 26 | 65.0 | 65.0 | 18 | 58.1 | 58.1 | 38 | 55.9 | 55.9 | 83 | 58.9 | 58.9 |
|  | other | 14 | 35.0 | 100.0 | 13 | 41.9 | 100.0 | 30 | 44.1 | 100.0 | 58 | 41.1 | 100.0 |
| p = 0.644 | all | 40 | 100.0 |  | 31 | 100.0 |  | 68 | 100.0 |  | 141 | 100.0 |  |
| B symptoms | No | 23 | 57.5 | 57.5 | 17 | 54.8 | 54.8 | 39 | 58.2 | 58.2 | 81 | 57.9 | 57.9 |
|  | Yes | 17 | 42.5 | 100.0 | 14 | 45.2 | 100.0 | 28 | 41.8 | 100.0 | 59 | 42.1 | 100.0 |
| p = 0.951 | all | 40 | 100.0 |  | 31 | 100.0 |  | 67 | 100.0 |  | 140 | 100.0 |  |
| Bulky mass | No | 32 | 80.0 | 80.0 | 26 | 83.9 | 83.9 | 52 | 76.5 | 76.5 | 112 | 79.4 | 79.4 |
|  | Yes | 8 | 20.0 | 100.0 | 5 | 16.1 | 100.0 | 16 | 23.5 | 100.0 | 29 | 20.6 | 100.0 |
| p = 0.693 | all | 40 | 100.0 |  | 31 | 100.0 |  | 68 | 100.0 |  | 141 | 100.0 |  |
| Anemia, Hb levels less than 10^5^ g/L | No | 34 | 85.0 | 85.0 | 23 | 74.2 | 74.2 | 51 | 75.0 | 75.0 | 110 | 78.0 | 78.0 |
|  | Yes | 6 | 15.0 | 100.0 | 8 | 25.8 | 100.0 | 17 | 25.0 | 100.0 | 31 | 22.0 | 100.0 |
| p = 0.420 | all | 40 | 100.0 |  | 31 | 100.0 |  | 68 | 100.0 |  | 141 | 100.0 |  |
| Leukocytosis, more than 15X10^9^/L | No | 35 | 87.5 | 87.5 | 28 | 93.3 | 93.3 | 58 | 85.3 | 85.3 | 123 | 87.9 | 87.9 |
|  | Yes | 5 | 12.5 | 100.0 | 2 | 6.7 | 100.0 | 10 | 14.7 | 100.0 | 17 | 12.1 | 100.0 |
| p = 0.536 | all | 40 | 100.0 |  | 30 | 100.0 |  | 68 | 100.0 |  | 140 | 100.0 |  |
| Lymphocytopenia, <0.6 X 10^9^/L or <8% of WBC | No | 36 | 90.0 | 90.0 | 24 | 80.0 | 80.0 | 59 | 89.4 | 89.4 | 121 | 87.7 | 87.7 |
|  | Yes | 4 | 10.0 | 100.0 | 6 | 20.0 | 100.0 | 7 | 10.6 | 100.0 | 17 | 12.3 | 100.0 |
| p = 0.370 | all | 40 | 100.0 |  | 30 | 100.0 |  | 66 | 100.0 |  | 138 | 100.0 |  |
| Hypoalbuminemia, <40 g/L | No | 24 | 68.6 | 68.6 | 20 | 66.7 | 66.7 | 41 | 63.1 | 63.1 | 86 | 65.2 | 65.2 |
|  | Yes | 11 | 31.4 | 100.0 | 10 | 33.3 | 100.0 | 24 | 36.9 | 100.0 | 46 | 34.9 | 100.0 |
| p = 0.847 | all | 35 | 100.0 |  | 30 | 100.0 |  | 65 | 100.0 |  | 132 | 100.0 |  |
| High LDH level, >450 UI/L | No | 29 | 72.5 | 72.5 | 19 | 61.3 | 61.3 | 49 | 74.2 | 74.2 | 99 | 71.2 | 71.2 |
|  | Yes | 11 | 27.5 | 100.0 | 12 | 38.7 | 100.0 | 17 | 25.8 | 100.0 | 40 | 28.8 | 100.0 |
| p = 0.409 | all | 40 | 100.0 |  | 31 | 100.0 |  | 66 | 100.0 |  | 139 | 100.0 |  |
| High B-2-microglobulin level, >25 mg/L | No | 25 | 80.7 | 80.7 | 16 | 69.6 | 69.6 | 41 | 74.5 | 74.5 | 84 | 75.7 | 75.7 |
|  | Yes | 6 | 19.4 | 100.0 | 7 | 30.4 | 100.0 | 14 | 25.4 | 100.0 | 27 | 24.3 | 100.0 |
| p = 0.638 | all | 31 | 100.0 |  | 23 | 100.0 |  | 55 | 100.0 |  | 111 | 100.0 |  |
| Stage | Early(I‐II) | 27 | 69.2 | 69.2 | 20 | 64.5 | 64.5 | 38 | 55.9 | 55.9 | 86 | 61.4 | 61.4 |
|  | Advanced (III‐IV) | 12 | 30.8 | 100.0 | 11 | 35.5 | 100.0 | 30 | 44.1 | 100.0 | 54 | 38.6 | 100.0 |
| p = 0.366 | all | 39 | 100.0 |  | 31 | 100.0 |  | 68 | 100.0 |  | 140 | 100.0 |  |
| Treatment | ABVD | 20 | 51.3 | 51.3 | 16 | 53.3 | 53.3 | 36 | 53.7 | 53.7 | 73 | 52.9 | 52.9 |
|  | MOPABV | 15 | 38.5 | 89.7 | 13 | 43.3 | 96.7 | 24 | 35.8 | 89.5 | 53 | 38.4 | 91.3 |
|  | MOPP | 3 | 7.7 | 97.4 | 1 | 3.3 | 100.0 | 4 | 6.0 | 95.5 | 8 | 5.8 | 97.1 |
|  | Other | 1 | 2.6 | 100.0 | 0 | 0.0 | 100.0 | 3 | 4.5 | 100.0 | 4 | 2.9 | 100.0 |
| p = 0.888 | all | 39 | 100.0 |  | 30 | 100.0 |  | 67 | 100.0 |  | 138 | 100.0 |  |
| EBV | No | 19 | 70.4 | 70.4 | 12 | 54.5 | 54.5 | 33 | 60.0 | 60.0 | 65 | 61.9 | 61.9 |
|  | Yes | 8 | 29.6 | 100.0 | 10 | 45.5 | 100.0 | 22 | 40.0 | 100.0 | 40 | 38.1 | 100.0 |
| p = 0.497 | all | 27 | 100.0 |  | 22 | 100.0 |  | 55 | 100.0 |  | 105 | 100.0 |  |
| Neutropenia toxicity | No | 28 | 70.0 | 70.0 | 21 | 67.7 | 67.7 | 43 | 63.2 | 63.2 | 94 | 66.7 | 66.7 |
|  | Yes | 12 | 30.0 | 100.0 | 10 | 32.3 | 100.0 | 25 | 36.8 | 100.0 | 47 | 33.3 | 100.0 |
| p = 0.756 | all | 40 | 100.0 |  | 31 | 100.0 |  | 68 | 100.0 |  | 141 | 100.0 |  |
| Anemia toxicity | No | 39 | 97.5 | 97.5 | 30 | 96.8 | 96.8 | 64 | 94.1 | 94.1 | 135 | 95.7 | 95.7 |
|  | Yes | 1 | 2.5 | 100.0 | 1 | 3.2 | 100.0 | 4 | 5.9 | 100.0 | 6 | 4.3 | 100.0 |
| p = 0.666 | all | 40 | 100.0 |  | 31 | 100.0 |  | 68 | 100.0 |  | 141 | 100.0 |  |
| Thrombocytopenia toxicity | No | 38 | 95.0 | 95.0 | 30 | 96.8 | 96.8 | 65 | 95.6 | 95.6 | 135 | 95.7 | 95.7 |
|  | Yes | 2 | 5.0 | 100.0 | 1 | 3.2 | 100.0 | 3 | 4.4 | 100.0 | 6 | 4.3 | 100.0 |
| p =0.934 | all | 40 | 100.0 |  | 31 | 100.0 |  | 68 | 100.0 |  | 141 | 100.0 |  |
| Pulmonary toxicity | No | 37 | 92.5 | 92.5 | 30 | 96.8 | 96.8 | 64 | 95.5 | 95.5 | 133 | 95.0 | 95.0 |
|  | Yes | 3 | 7.5 | 100.0 | 1 | 3.2 | 100.0 | 3 | 4.5 | 100.0 | 7 | 5.0 | 100.0 |
| p = 0.684 | all | 40 | 100.0 |  | 31 | 100.0 |  | 67 | 100.0 |  | 140 | 100.0 |  |
| Neurological toxicity | No | 34 | 85.0 | 85.0 | 26 | 83.9 | 83.9 | 57 | 83.8 | 83.8 | 118 | 83.7 | 83.7 |
|  | Yes | 6 | 15.0 | 100.0 | 5 | 16.1 | 100.0 | 11 | 16.2 | 100.0 | 23 | 16.3 | 100.0 |
| p = 0.986 | all | 40 | 100.0 |  | 31 | 100.0 |  | 68 | 100.0 |  | 141 | 100.0 |  |
| Infectious toxicity | No | 27 | 67.5 | 67.5 | 16 | 51.6 | 51.6 | 45 | 66.2 | 66.2 | 90 | 63.8 | 63.8 |
|  | Yes | 13 | 32.5 | 100.0 | 15 | 48.4 | 100.0 | 23 | 33.8 | 100.0 | 51 | 36.2 | 100.0 |
| p = 0.306 | all | 40 | 100.0 |  | 31 | 100.0 |  | 68 | 100.0 |  | 141 | 100.0 |  |

1. **TRBP combination (1🡪CC; 2🡪TT+TC).**

| **Variable** | **Levels** | **N_1_** | **%_1_** | **∑%_1_** | **N_2_** | **%_2_** | **∑%_2_** | **N_all_** | **%_all_** | **∑%_all_** |
| --- | --- | --- | --- | --- | --- | --- | --- | --- | --- | --- |
| Sex | female | 24 | 60.0 | 60.0 | 43 | 43.4 | 43.4 | 69 | 48.9 | 48.9 |
|  | male | 16 | 40.0 | 100.0 | 56 | 56.6 | 100.0 | 72 | 51.1 | 100.0 |
| p = 0.093 | all | 40 | 100.0 |  | 99 | 100.0 |  | 141 | 100.0 |  |
| Age | ¡45 | 35 | 87.5 | 87.5 | 74 | 74.8 | 74.8 | 110 | 78.0 | 78.0 |
|  | ≥45 | 5 | 12.5 | 100.0 | 25 | 25.2 | 100.0 | 31 | 22.0 | 100.0 |
| p = 0.115 | all | 40 | 100.0 |  | 99 | 100.0 |  | 141 | 100.0 |  |
| Histology | EN | 26 | 65.0 | 65.0 | 56 | 56.6 | 56.6 | 83 | 58.9 | 58.9 |
|  | other | 14 | 35.0 | 100.0 | 43 | 43.4 | 100.0 | 58 | 41.1 | 100.0 |
| p = 0.447 | all | 40 | 100.0 |  | 99 | 100.0 |  | 141 | 100.0 |  |
| B symptoms | No | 23 | 57.5 | 57.5 | 56 | 57.1 | 57.1 | 81 | 57.9 | 57.9 |
|  | Yes | 17 | 42.5 | 100.0 | 42 | 42.9 | 100.0 | 59 | 42.1 | 100.0 |
| p = 1.000 | all | 40 | 100.0 |  | 98 | 100.0 |  | 140 | 100.0 |  |
| Bulky mass | No | 32 | 80.0 | 80.0 | 78 | 78.8 | 78.8 | 112 | 79.4 | 79.4 |
|  | Yes | 8 | 20.0 | 100.0 | 21 | 21.2 | 100.0 | 29 | 20.6 | 100.0 |
| p = 1.000 | all | 40 | 100.0 |  | 99 | 100.0 |  | 141 | 100.0 |  |
| Anemia, Hb levels less than 10^5^ g/L | No | 34 | 85.0 | 85.0 | 74 | 74.8 | 74.8 | 110 | 78.0 | 78.0 |
|  | Yes | 6 | 15.0 | 100.0 | 25 | 25.2 | 100.0 | 31 | 22.0 | 100.0 |
| p = 0.261 | all | 40 | 100.0 |  | 99 | 100.0 |  | 141 | 100.0 |  |
| Leukocytosis, more than 15X10^9^/L | No | 35 | 87.5 | 87.5 | 86 | 87.8 | 87.8 | 123 | 87.9 | 87.9 |
|  | Yes | 5 | 12.5 | 100.0 | 12 | 12.2 | 100.0 | 17 | 12.1 | 100.0 |
| p = 1.000 | all | 40 | 100.0 |  | 98 | 100.0 |  | 140 | 100.0 |  |
| Lymphocytopenia, <0.6 X 10^9^/L or <8% of WBC | No | 36 | 90.0 | 90.0 | 83 | 86.5 | 86.5 | 121 | 87.7 | 87.7 |
|  | Yes | 4 | 10.0 | 100.0 | 13 | 13.5 | 100.0 | 17 | 12.3 | 100.0 |
| p = 0.777 | all | 40 | 100.0 |  | 96 | 100.0 |  | 138 | 100.0 |  |
| Hypoalbuminemia, <40 g/L | No | 24 | 68.6 | 68.6 | 61 | 64.2 | 64.2 | 86 | 65.2 | 65.2 |
|  | Yes | 11 | 31.4 | 100.0 | 34 | 35.8 | 100.0 | 46 | 34.9 | 100.0 |
| p = 0.683 | all | 35 | 100.0 |  | 95 | 100.0 |  | 132 | 100.0 |  |
| High LDH level, >450 UI/L | No | 29 | 72.5 | 72.5 | 68 | 70.1 | 70.1 | 99 | 71.2 | 71.2 |
|  | Yes | 11 | 27.5 | 100.0 | 29 | 29.9 | 100.0 | 40 | 28.8 | 100.0 |
| p = 0.839 | all | 40 | 100.0 |  | 97 | 100.0 |  | 139 | 100.0 |  |
| High B-2-microglobulin level, >25 mg/L | No | 25 | 80.7 | 80.7 | 57 | 73.1 | 73.1 | 84 | 75.7 | 75.7 |
|  | Yes | 6 | 19.4 | 100.0 | 21 | 26.9 | 100.0 | 27 | 24.3 | 100.0 |
| p = 0.470 | all | 31 | 100.0 |  | 78 | 100.0 |  | 111 | 100.0 |  |
| Stage | Early(I‐II) | 27 | 69.2 | 69.2 | 58 | 58.6 | 58.6 | 86 | 61.4 | 61.4 |
|  | Advanced (III‐IV) | 12 | 30.8 | 100.0 | 41 | 41.4 | 100.0 | 54 | 38.6 | 100.0 |
| p = 0.331 | all | 39 | 100.0 |  | 99 | 100.0 |  | 140 | 100.0 |  |
| Treatment | ABVD | 20 | 51.3 | 51.3 | 52 | 53.6 | 53.6 | 73 | 52.9 | 52.9 |
|  | MOPABV | 15 | 38.5 | 89.7 | 37 | 38.1 | 91.8 | 53 | 38.4 | 91.3 |
|  | MOPP | 3 | 7.7 | 97.4 | 5 | 5.2 | 96.9 | 8 | 5.8 | 97.1 |
|  | Other | 1 | 2.6 | 100.0 | 3 | 3.1 | 100.0 | 4 | 2.9 | 100.0 |
| p = 0.948 | all | 39 | 100.0 |  | 97 | 100.0 |  | 138 | 100.0 |  |
| EBV | No | 19 | 70.4 | 70.4 | 45 | 58.4 | 58.4 | 65 | 61.9 | 61.9 |
|  | Yes | 8 | 29.6 | 100.0 | 32 | 41.6 | 100.0 | 40 | 38.1 | 100.0 |
| p = 0.359 | all | 27 | 100.0 |  | 77 | 100.0 |  | 105 | 100.0 |  |
| Neutropenia toxicity | No | 28 | 70.0 | 70.0 | 64 | 64.7 | 64.7 | 94 | 66.7 | 66.7 |
|  | Yes | 12 | 30.0 | 100.0 | 35 | 35.4 | 100.0 | 47 | 33.3 | 100.0 |
| p = 0.692 | all | 40 | 100.0 |  | 99 | 100.0 |  | 141 | 100.0 |  |
| Anemia toxicity | No | 39 | 97.5 | 97.5 | 94 | 95.0 | 95.0 | 135 | 95.7 | 95.7 |
|  | Yes | 1 | 2.5 | 100.0 | 5 | 5.0 | 100.0 | 6 | 4.3 | 100.0 |
| p = 0.673 | all | 40 | 100.0 |  | 99 | 100.0 |  | 141 | 100.0 |  |
| Thrombocytopenia toxicity | No | 38 | 95.0 | 95.0 | 95 | 96.0 | 96.0 | 135 | 95.7 | 95.7 |
|  | Yes | 2 | 5.0 | 100.0 | 4 | 4.0 | 100.0 | 6 | 4.3 | 100.0 |
| p = 1.000 | all | 40 | 100.0 |  | 99 | 100.0 |  | 141 | 100.0 |  |
| Pulmonary toxicity | No | 37 | 92.5 | 92.5 | 94 | 95.9 | 95.9 | 133 | 95.0 | 95.0 |
|  | Yes | 3 | 7.5 | 100.0 | 4 | 4.1 | 100.0 | 7 | 5.0 | 100.0 |
| p = 0.413 | all | 40 | 100.0 |  | 98 | 100.0 |  | 140 | 100.0 |  |
| Neurological toxicity | No | 34 | 85.0 | 85.0 | 83 | 83.8 | 83.8 | 118 | 83.7 | 83.7 |
|  | Yes | 6 | 15.0 | 100.0 | 16 | 16.2 | 100.0 | 23 | 16.3 | 100.0 |
| p = 1.000 | all | 40 | 100.0 |  | 99 | 100.0 |  | 141 | 100.0 |  |
| Infectious toxicity | No | 27 | 67.5 | 67.5 | 61 | 61.6 | 61.6 | 90 | 63.8 | 63.8 |
|  | Yes | 13 | 32.5 | 100.0 | 38 | 38.4 | 100.0 | 51 | 36.2 | 100.0 |
| p = 0.564 | all | 40 | 100.0 |  | 99 | 100.0 |  | 141 | 100.0 |  |

1. **XPO5 combination (1🡪AC; 2🡪AA+CC).**

[ht]

| **Variable** | **Levels** | **N_1_** | **%_1_** | **∑%_1_** | **N_2_** | **%_2_** | **∑%_2_** | **N_all_** | **%_all_** | **∑%_all_** |
| --- | --- | --- | --- | --- | --- | --- | --- | --- | --- | --- |
| Sex | female | 32 | 45.1 | 45.1 | 31 | 51.7 | 51.7 | 69 | 48.9 | 48.9 |
|  | male | 39 | 54.9 | 100.0 | 29 | 48.3 | 100.0 | 72 | 51.1 | 100.0 |
| p = 0.486 | all | 71 | 100.0 |  | 60 | 100.0 |  | 141 | 100.0 |  |
| Age | <45 | 53 | 74.7 | 74.7 | 48 | 80.0 | 80.0 | 110 | 78.0 | 78.0 |
|  | ≥45 | 18 | 25.4 | 100.0 | 12 | 20.0 | 100.0 | 31 | 22.0 | 100.0 |
| p = 0.534 | all | 71 | 100.0 |  | 60 | 100.0 |  | 141 | 100.0 |  |
| Histology | EN | 42 | 59.1 | 59.1 | 33 | 55.0 | 55.0 | 83 | 58.9 | 58.9 |
|  | other | 29 | 40.9 | 100.0 | 27 | 45.0 | 100.0 | 58 | 41.1 | 100.0 |
| p = 0.723 | all | 71 | 100.0 |  | 60 | 100.0 |  | 141 | 100.0 |  |
| B symptoms | No | 44 | 62.9 | 62.9 | 30 | 50.0 | 50.0 | 81 | 57.9 | 57.9 |
|  | Yes | 26 | 37.1 | 100.0 | 30 | 50.0 | 100.0 | 59 | 42.1 | 100.0 |
| p = 0.158 | all | 70 | 100.0 |  | 60 | 100.0 |  | 140 | 100.0 |  |
| Bulky mass | No | 65 | 91.5 | 91.5 | 42 | 70.0 | 70.0 | 112 | 79.4 | 79.4 |
|  | Yes | 6 | 8.4 | 100.0 | 18 | 30.0 | 100.0 | 29 | 20.6 | 100.0 |
| p = 0.003 | all | 71 | 100.0 |  | 60 | 100.0 |  | 141 | 100.0 |  |
| Anemia, Hb levels less than 10^5^ g/L | No | 58 | 81.7 | 81.7 | 43 | 71.7 | 71.7 | 110 | 78.0 | 78.0 |
|  | Yes | 13 | 18.3 | 100.0 | 17 | 28.3 | 100.0 | 31 | 22.0 | 100.0 |
| p = 0.212 | all | 71 | 100.0 |  | 60 | 100.0 |  | 141 | 100.0 |  |
| Leukocytosis, more than 15X10^9^/L | No | 62 | 88.6 | 88.6 | 52 | 86.7 | 86.7 | 123 | 87.9 | 87.9 |
|  | Yes | 8 | 11.4 | 100.0 | 8 | 13.3 | 100.0 | 17 | 12.1 | 100.0 |
| p = 0.793 | all | 70 | 100.0 |  | 60 | 100.0 |  | 140 | 100.0 |  |
| Lymphocytopenia, <0.6 X 10^9^/L or <8% of WBC | No | 63 | 92.7 | 92.7 | 48 | 80.0 | 80.0 | 121 | 87.7 | 87.7 |
|  | Yes | 5 | 7.3 | 100.0 | 12 | 20.0 | 100.0 | 17 | 12.3 | 100.0 |
| p = 0.041 | all | 68 | 100.0 |  | 60 | 100.0 |  | 138 | 100.0 |  |
| Hypoalbuminemia, <40 g/L | No | 45 | 70.3 | 70.3 | 37 | 63.8 | 63.8 | 86 | 65.2 | 65.2 |
|  | Yes | 19 | 29.7 | 100.0 | 21 | 36.2 | 100.0 | 46 | 34.9 | 100.0 |
| p = 0.563 | all | 64 | 100.0 |  | 58 | 100.0 |  | 132 | 100.0 |  |
| High LDH level, >450 UI/L | No | 52 | 75.4 | 75.4 | 40 | 66.7 | 66.7 | 99 | 71.2 | 71.2 |
|  | Yes | 17 | 24.6 | 100.0 | 20 | 33.3 | 100.0 | 40 | 28.8 | 100.0 |
| p = 0.331 | all | 69 | 100.0 |  | 60 | 100.0 |  | 139 | 100.0 |  |
| High B-2-microglobulin level, >25 mg/L | No | 43 | 79.6 | 79.6 | 33 | 68.8 | 68.8 | 84 | 75.7 | 75.7 |
|  | Yes | 11 | 20.4 | 100.0 | 15 | 31.2 | 100.0 | 27 | 24.3 | 100.0 |
| p = 0.257 | all | 54 | 100.0 |  | 48 | 100.0 |  | 111 | 100.0 |  |
| Stage | Early(I‐II) | 47 | 66.2 | 66.2 | 31 | 52.5 | 52.5 | 86 | 61.4 | 61.4 |
|  | Advanced (III‐IV) | 24 | 33.8 | 100.0 | 28 | 47.5 | 100.0 | 54 | 38.6 | 100.0 |
| p = 0.150 | all | 71 | 100.0 |  | 59 | 100.0 |  | 140 | 100.0 |  |
| Treatment | ABVD | 43 | 62.3 | 62.3 | 30 | 50.9 | 50.9 | 73 | 52.9 | 52.9 |
|  | MOPABV | 19 | 27.5 | 89.9 | 25 | 42.4 | 93.2 | 53 | 38.4 | 91.3 |
|  | MOPP | 5 | 7.2 | 97.1 | 2 | 3.4 | 96.6 | 8 | 5.8 | 97.1 |
|  | Other | 2 | 2.9 | 100.0 | 2 | 3.4 | 100.0 | 4 | 2.9 | 100.0 |
| p = 0.300 | all | 69 | 100.0 |  | 59 | 100.0 |  | 138 | 100.0 |  |
| EBV | No | 36 | 66.7 | 66.7 | 27 | 55.1 | 55.1 | 65 | 61.9 | 61.9 |
|  | Yes | 18 | 33.3 | 100.0 | 22 | 44.9 | 100.0 | 40 | 38.1 | 100.0 |
| p = 0.312 | all | 54 | 100.0 |  | 49 | 100.0 |  | 105 | 100.0 |  |
| Neutropenia toxicity | No | 46 | 64.8 | 64.8 | 40 | 66.7 | 66.7 | 94 | 66.7 | 66.7 |
|  | Yes | 25 | 35.2 | 100.0 | 20 | 33.3 | 100.0 | 47 | 33.3 | 100.0 |
| p = 0.885 | all | 71 | 100.0 |  | 60 | 100.0 |  | 141 | 100.0 |  |
| Anemia toxicity | No | 68 | 95.8 | 95.8 | 57 | 95.0 | 95.0 | 135 | 95.7 | 95.7 |
|  | Yes | 3 | 4.2 | 100.0 | 3 | 5.0 | 100.0 | 6 | 4.3 | 100.0 |
| p = 1.000 | all | 71 | 100.0 |  | 60 | 100.0 |  | 141 | 100.0 |  |
| Thrombocytopenia toxicity | No | 69 | 97.2 | 97.2 | 57 | 95.0 | 95.0 | 135 | 95.7 | 95.7 |
|  | Yes | 2 | 2.8 | 100.0 | 3 | 5.0 | 100.0 | 6 | 4.3 | 100.0 |
| p = 0.660 | all | 71 | 100.0 |  | 60 | 100.0 |  | 141 | 100.0 |  |
| Pulmonary toxicity | No | 69 | 98.6 | 98.6 | 54 | 90.0 | 90.0 | 133 | 95.0 | 95.0 |
|  | Yes | 1 | 1.4 | 100.0 | 6 | 10.0 | 100.0 | 7 | 5.0 | 100.0 |
| p = 0.048 | all | 70 | 100.0 |  | 60 | 100.0 |  | 140 | 100.0 |  |
| Neurological toxicity | No | 64 | 90.1 | 90.1 | 49 | 81.7 | 81.7 | 118 | 83.7 | 83.7 |
|  | Yes | 7 | 9.9 | 100.0 | 11 | 18.3 | 100.0 | 23 | 16.3 | 100.0 |
| p = 0.205 | all | 71 | 100.0 |  | 60 | 100.0 |  | 141 | 100.0 |  |
| Infectious toxicity | No | 42 | 59.1 | 59.1 | 41 | 68.3 | 68.3 | 90 | 63.8 | 63.8 |
|  | Yes | 29 | 40.9 | 100.0 | 19 | 31.7 | 100.0 | 51 | 36.2 | 100.0 |
| p = 0.363 | all | 71 | 100.0 |  | 60 | 100.0 |  | 141 | 100.0 |  |

1. **MIR196A2 combination (1🡪CC; 2🡪CT+TT).**

| **Variable** | **Levels** | **N_1_** | **%_1_** | **∑%_1_** | **N_2_** | **%_2_** | **∑%_2_** | **N_all_** | **%_all_** | **∑%_all_** |
| --- | --- | --- | --- | --- | --- | --- | --- | --- | --- | --- |
| Sex | female | 36 | 59.0 | 59.0 | 29 | 38.2 | 38.2 | 69 | 48.9 | 48.9 |
|  | male | 25 | 41.0 | 100.0 | 47 | 61.8 | 100.0 | 72 | 51.1 | 100.0 |
| p = 0.017 | all | 61 | 100.0 |  | 76 | 100.0 |  | 141 | 100.0 |  |
| Age | <45 | 48 | 78.7 | 78.7 | 59 | 77.6 | 77.6 | 110 | 78.0 | 78.0 |
|  | ≥45 | 13 | 21.3 | 100.0 | 17 | 22.4 | 100.0 | 31 | 22.0 | 100.0 |
| p = 1.000 | all | 61 | 100.0 |  | 76 | 100.0 |  | 141 | 100.0 |  |
| Histology | EN | 43 | 70.5 | 70.5 | 38 | 50.0 | 50.0 | 83 | 58.9 | 58.9 |
|  | other | 18 | 29.5 | 100.0 | 38 | 50.0 | 100.0 | 58 | 41.1 | 100.0 |
| p = 0.023 | all | 61 | 100.0 |  | 76 | 100.0 |  | 141 | 100.0 |  |
| B symptoms | No | 40 | 65.6 | 65.6 | 37 | 49.3 | 49.3 | 81 | 57.9 | 57.9 |
|  | Yes | 21 | 34.4 | 100.0 | 38 | 50.7 | 100.0 | 59 | 42.1 | 100.0 |
| p = 0.082 | all | 61 | 100.0 |  | 75 | 100.0 |  | 140 | 100.0 |  |
| Bulky mass | No | 43 | 70.5 | 70.5 | 67 | 88.2 | 88.2 | 112 | 79.4 | 79.4 |
|  | Yes | 18 | 29.5 | 100.0 | 9 | 11.8 | 100.0 | 29 | 20.6 | 100.0 |
| p = 0.016 | all | 61 | 100.0 |  | 76 | 100.0 |  | 141 | 100.0 |  |
| Anemia, Hb levels less than 10^5^ g/L | No | 49 | 80.3 | 80.3 | 57 | 75.0 | 75.0 | 110 | 78.0 | 78.0 |
|  | Yes | 12 | 19.7 | 100.0 | 19 | 25.0 | 100.0 | 31 | 22.0 | 100.0 |
| p = 0.540 | all | 61 | 100.0 |  | 76 | 100.0 |  | 141 | 100.0 |  |
| Leukocytosis, more than 15X10^9^/L | No | 49 | 81.7 | 81.7 | 70 | 92.1 | 92.1 | 123 | 87.9 | 87.9 |
|  | Yes | 11 | 18.3 | 100.0 | 6 | 7.9 | 100.0 | 17 | 12.1 | 100.0 |
| p = 0.115 | all | 60 | 100.0 |  | 76 | 100.0 |  | 140 | 100.0 |  |
| Lymphocytopenia, <0.6 X 10^9^/L or <8% of WBC | No | 56 | 93.3 | 93.3 | 61 | 82.4 | 82.4 | 121 | 87.7 | 87.7 |
|  | Yes | 4 | 6.7 | 100.0 | 13 | 17.6 | 100.0 | 17 | 12.3 | 100.0 |
| p = 0.071 | all | 60 | 100.0 |  | 74 | 100.0 |  | 138 | 100.0 |  |
| Hypoalbuminemia, <40 g/L | No | 43 | 74.1 | 74.1 | 40 | 57.1 | 57.1 | 86 | 65.2 | 65.2 |
|  | Yes | 15 | 25.9 | 100.0 | 30 | 42.9 | 100.0 | 46 | 34.9 | 100.0 |
| p = 0.063 | all | 58 | 100.0 |  | 70 | 100.0 |  | 132 | 100.0 |  |
| High LDH level, >450 UI/L | No | 42 | 70.0 | 70.0 | 53 | 70.7 | 70.7 | 99 | 71.2 | 71.2 |
|  | Yes | 18 | 30.0 | 100.0 | 22 | 29.3 | 100.0 | 40 | 28.8 | 100.0 |
| p = 1.000 | all | 60 | 100.0 |  | 75 | 100.0 |  | 139 | 100.0 |  |
| High B-2-microglobulin level, >25 mg/L | No | 35 | 79.5 | 79.5 | 45 | 71.4 | 71.4 | 84 | 75.7 | 75.7 |
|  | Yes | 9 | 20.4 | 100.0 | 18 | 28.6 | 100.0 | 27 | 24.3 | 100.0 |
| p = 0.375 | all | 44 | 100.0 |  | 63 | 100.0 |  | 111 | 100.0 |  |
| Stage | Early(I‐II) | 39 | 63.9 | 63.9 | 43 | 57.3 | 57.3 | 86 | 61.4 | 61.4 |
|  | Advanced (III‐IV) | 22 | 36.1 | 100.0 | 32 | 42.7 | 100.0 | 54 | 38.6 | 100.0 |
| p = 0.483 | all | 61 | 100.0 |  | 75 | 100.0 |  | 140 | 100.0 |  |
| Treatment | ABVD | 32 | 52.5 | 52.5 | 40 | 54.8 | 54.8 | 73 | 52.9 | 52.9 |
|  | MOPABV | 25 | 41.0 | 93.4 | 25 | 34.2 | 89.0 | 53 | 38.4 | 91.3 |
|  | MOPP | 3 | 4.9 | 98.4 | 5 | 6.8 | 95.9 | 8 | 5.8 | 97.1 |
|  | Other | 1 | 1.6 | 100.0 | 3 | 4.1 | 100.0 | 4 | 2.9 | 100.0 |
| p = 0.723 | all | 61 | 100.0 |  | 73 | 100.0 |  | 138 | 100.0 |  |
| EBV | No | 31 | 67.4 | 67.4 | 33 | 56.9 | 56.9 | 65 | 61.9 | 61.9 |
|  | Yes | 15 | 32.6 | 100.0 | 25 | 43.1 | 100.0 | 40 | 38.1 | 100.0 |
| p = 0.314 | all | 46 | 100.0 |  | 58 | 100.0 |  | 105 | 100.0 |  |
| Neutropenia toxicity | No | 32 | 59.3 | 59.3 | 47 | 70.2 | 70.2 | 94 | 66.7 | 66.7 |
|  | Yes | 22 | 40.7 | 100.0 | 20 | 29.9 | 100.0 | 47 | 33.3 | 100.0 |
| p = 0.251 | all | 54 | 100.0 |  | 67 | 100.0 |  | 141 | 100.0 |  |
| Anemia toxicity | No | 51 | 94.4 | 94.4 | 64 | 95.5 | 95.5 | 135 | 95.7 | 95.7 |
|  | Yes | 3 | 5.6 | 100.0 | 3 | 4.5 | 100.0 | 6 | 4.3 | 100.0 |
| p = 1.000 | all | 54 | 100.0 |  | 67 | 100.0 |  | 141 | 100.0 |  |
| Thrombocytopenia toxicity | No | 51 | 94.4 | 94.4 | 65 | 97.0 | 97.0 | 135 | 95.7 | 95.7 |
|  | Yes | 3 | 5.6 | 100.0 | 2 | 3.0 | 100.0 | 6 | 4.3 | 100.0 |
| p = 0.655 | all | 54 | 100.0 |  | 67 | 100.0 |  | 141 | 100.0 |  |
| Pulmonary toxicity | No | 53 | 98.2 | 98.2 | 61 | 92.4 | 92.4 | 133 | 95.0 | 95.0 |
|  | Yes | 1 | 1.9 | 100.0 | 5 | 7.6 | 100.0 | 7 | 5.0 | 100.0 |
| p = 0.221 | all | 54 | 100.0 |  | 66 | 100.0 |  | 140 | 100.0 |  |
| Neurological toxicity | No | 46 | 85.2 | 85.2 | 59 | 88.1 | 88.1 | 118 | 83.7 | 83.7 |
|  | Yes | 8 | 14.8 | 100.0 | 8 | 11.9 | 100.0 | 23 | 16.3 | 100.0 |
| p = 0.788 | all | 54 | 100.0 |  | 67 | 100.0 |  | 141 | 100.0 |  |
| Infectious toxicity | No | 31 | 57.4 | 57.4 | 45 | 67.2 | 67.2 | 90 | 63.8 | 63.8 |
|  | Yes | 23 | 42.6 | 100.0 | 22 | 32.8 | 100.0 | 51 | 36.2 | 100.0 |
| p = 0.344 | all | 54 | 100.0 |  | 67 | 100.0 |  | 141 | 100.0 |  |

1. **Combination of TRBP and XPO5 (1🡪 XPO5 AA/CC + TRBP TT/TC; 2🡪Other combinations).**

| **Variable** | **Levels** | **N_1_** | **%_1_** | **∑%_1_** | **N_2_** | **%_2_** | **∑%_2_** | **N_all_** | **%_all_** | **∑%_all_** |
| --- | --- | --- | --- | --- | --- | --- | --- | --- | --- | --- |
| Sex | female | 21 | 46.7 | 46.7 | 42 | 48.8 | 48.8 | 69 | 48.9 | 48.9 |
|  | male | 24 | 53.3 | 100.0 | 44 | 51.2 | 100.0 | 72 | 51.1 | 100.0 |
| p = 0.855 | all | 45 | 100.0 |  | 86 | 100.0 |  | 141 | 100.0 |  |
| Age | <45 | 35 | 77.8 | 77.8 | 66 | 76.7 | 76.7 | 110 | 78.0 | 78.0 |
|  | ≥45 | 10 | 22.2 | 100.0 | 20 | 23.3 | 100.0 | 31 | 22.0 | 100.0 |
| p = 1.000 | all | 45 | 100.0 |  | 86 | 100.0 |  | 141 | 100.0 |  |
| Histology | EN | 24 | 53.3 | 53.3 | 51 | 59.3 | 59.3 | 83 | 58.9 | 58.9 |
|  | other | 21 | 46.7 | 100.0 | 35 | 40.7 | 100.0 | 58 | 41.1 | 100.0 |
| p = 0.578 | all | 45 | 100.0 |  | 86 | 100.0 |  | 141 | 100.0 |  |
| B symptoms | No | 22 | 48.9 | 48.9 | 52 | 61.2 | 61.2 | 81 | 57.9 | 57.9 |
|  | Yes | 23 | 51.1 | 100.0 | 33 | 38.8 | 100.0 | 59 | 42.1 | 100.0 |
| p = 0.197 | all | 45 | 100.0 |  | 85 | 100.0 |  | 140 | 100.0 |  |
| Bulky mass | No | 31 | 68.9 | 68.9 | 76 | 88.4 | 88.4 | 112 | 79.4 | 79.4 |
|  | Yes | 14 | 31.1 | 100.0 | 10 | 11.6 | 100.0 | 29 | 20.6 | 100.0 |
| p = 0.009 | all | 45 | 100.0 |  | 86 | 100.0 |  | 141 | 100.0 |  |
| Anemia, Hb levels less than 10^5^ g/L | No | 31 | 68.9 | 68.9 | 70 | 81.4 | 81.4 | 110 | 78.0 | 78.0 |
|  | Yes | 14 | 31.1 | 100.0 | 16 | 18.6 | 100.0 | 31 | 22.0 | 100.0 |
| p = 0.127 | all | 45 | 100.0 |  | 86 | 100.0 |  | 141 | 100.0 |  |
| Leukocytosis, more than 15X10^9^/L | No | 38 | 84.4 | 84.4 | 76 | 89.4 | 89.4 | 123 | 87.9 | 87.9 |
|  | Yes | 7 | 15.6 | 100.0 | 9 | 10.6 | 100.0 | 17 | 12.1 | 100.0 |
| p = 0.414 | all | 45 | 100.0 |  | 85 | 100.0 |  | 140 | 100.0 |  |
| Lymphocytopenia, <0.6 X 10^9^/L or <8% of WBC | No | 36 | 80.0 | 80.0 | 75 | 90.4 | 90.4 | 121 | 87.7 | 87.7 |
|  | Yes | 9 | 20.0 | 100.0 | 8 | 9.6 | 100.0 | 17 | 12.3 | 100.0 |
| p = 0.110 | all | 45 | 100.0 |  | 83 | 100.0 |  | 138 | 100.0 |  |
| Hypoalbuminemia, <40 g/L | No | 29 | 64.4 | 64.4 | 53 | 68.8 | 68.8 | 86 | 65.2 | 65.2 |
|  | Yes | 16 | 35.6 | 100.0 | 24 | 31.2 | 100.0 | 46 | 34.9 | 100.0 |
| p = 0.691 | all | 45 | 100.0 |  | 77 | 100.0 |  | 132 | 100.0 |  |
| High LDH level, >450 UI/L | No | 29 | 64.4 | 64.4 | 63 | 75.0 | 75.0 | 99 | 71.2 | 71.2 |
|  | Yes | 16 | 35.6 | 100.0 | 21 | 25.0 | 100.0 | 40 | 28.8 | 100.0 |
| p = 0.225 | all | 45 | 100.0 |  | 84 | 100.0 |  | 139 | 100.0 |  |
| High B-2-microglobulin level, >25 mg/L | No | 24 | 68.6 | 68.6 | 52 | 77.6 | 77.6 | 84 | 75.7 | 75.7 |
|  | Yes | 11 | 31.4 | 100.0 | 15 | 22.4 | 100.0 | 27 | 24.3 | 100.0 |
| p = 0.346 | all | 35 | 100.0 |  | 67 | 100.0 |  | 111 | 100.0 |  |
| Stage | Early(I‐II) | 23 | 51.1 | 51.1 | 55 | 64.7 | 64.7 | 86 | 61.4 | 61.4 |
|  | Advanced (III‐IV) | 22 | 48.9 | 100.0 | 30 | 35.3 | 100.0 | 54 | 38.6 | 100.0 |
| p = 0.138 | all | 45 | 100.0 |  | 85 | 100.0 |  | 140 | 100.0 |  |
| Treatment | ABVD | 22 | 50.0 | 50.0 | 51 | 60.7 | 60.7 | 73 | 52.9 | 52.9 |
|  | MOPABV | 20 | 45.5 | 95.5 | 24 | 28.6 | 89.3 | 53 | 38.4 | 91.3 |
|  | MOPP | 1 | 2.3 | 97.7 | 6 | 7.1 | 96.4 | 8 | 5.8 | 97.1 |
|  | Other | 1 | 2.3 | 100.0 | 3 | 3.6 | 100.0 | 4 | 2.9 | 100.0 |
| p = 0.223 | all | 44 | 100.0 |  | 84 | 100.0 |  | 138 | 100.0 |  |
| EBV | No | 19 | 52.8 | 52.8 | 44 | 65.7 | 65.7 | 65 | 61.9 | 61.9 |
|  | Yes | 17 | 47.2 | 100.0 | 23 | 34.3 | 100.0 | 40 | 38.1 | 100.0 |
| p = 0.212 | all | 36 | 100.0 |  | 67 | 100.0 |  | 105 | 100.0 |  |
| Neutropenia toxicity | No | 29 | 64.4 | 64.4 | 57 | 66.3 | 66.3 | 94 | 66.7 | 66.7 |
|  | Yes | 16 | 35.6 | 100.0 | 29 | 33.7 | 100.0 | 47 | 33.3 | 100.0 |
| p = 0.848 | all | 45 | 100.0 |  | 86 | 100.0 |  | 141 | 100.0 |  |
| Anemia toxicity | No | 43 | 95.6 | 95.6 | 82 | 95.3 | 95.3 | 135 | 95.7 | 95.7 |
|  | Yes | 2 | 4.4 | 100.0 | 4 | 4.7 | 100.0 | 6 | 4.3 | 100.0 |
| p = 1.000 | all | 45 | 100.0 |  | 86 | 100.0 |  | 141 | 100.0 |  |
| Thrombocytopenia toxicity | No | 43 | 95.6 | 95.6 | 83 | 96.5 | 96.5 | 135 | 95.7 | 95.7 |
|  | Yes | 2 | 4.4 | 100.0 | 3 | 3.5 | 100.0 | 6 | 4.3 | 100.0 |
| p = 1.000 | all | 45 | 100.0 |  | 86 | 100.0 |  | 141 | 100.0 |  |
| Pulmonary toxicity | No | 42 | 93.3 | 93.3 | 81 | 95.3 | 95.3 | 133 | 95.0 | 95.0 |
|  | Yes | 3 | 6.7 | 100.0 | 4 | 4.7 | 100.0 | 7 | 5.0 | 100.0 |
| p = 0.692 | all | 45 | 100.0 |  | 85 | 100.0 |  | 140 | 100.0 |  |
| Neurological toxicity | No | 37 | 82.2 | 82.2 | 76 | 88.4 | 88.4 | 118 | 83.7 | 83.7 |
|  | Yes | 8 | 17.8 | 100.0 | 10 | 11.6 | 100.0 | 23 | 16.3 | 100.0 |
| p = 0.423 | all | 45 | 100.0 |  | 86 | 100.0 |  | 141 | 100.0 |  |
| Infectious toxicity | No | 29 | 64.4 | 64.4 | 54 | 62.8 | 62.8 | 90 | 63.8 | 63.8 |
|  | Yes | 16 | 35.6 | 100.0 | 32 | 37.2 | 100.0 | 51 | 36.2 | 100.0 |
| p = 1.000 | all | 45 | 100.0 |  | 86 | 100.0 |  | 141 | 100.0 |  |
