# Supplementary material for: MiR-SNPs as Markers of Toxicity and Clinical Outcome in Hodgkin Lymphoma Patients
Source: PLoS One. 2013 May 21;8(5):e64716. doi: 10.1371/journal.pone.0064716 (PMC3660374; doi:10.1371/journal.pone.0064716)
Supplement: Table S3 — Age adjusted analysis (Age<45 and age≥45). (DOCX) [file pone.0064716.s004.docx]

**Table S3.** Age adjusted analysis (Age<45 and age≥45).

| **Variable** | **Levels** | **N_<45_** | **%_<45_** | **∑%_<45_** | **N**_≥45_ | **%**_≥45_ | **∑%**_≥45_ | **DFS_<45_**  **_p-value_** | **DFS**_≥45_  **_p-value_** | **OS_<45_**  **_p-value_** | **OS**_≥45_  **_p-value_** |
| --- | --- | --- | --- | --- | --- | --- | --- | --- | --- | --- | --- |
| Sex | female | 52 | 47.3 | 47.3 | 17 | 54.8 | 54.8 | 0.9 | 0.2 | 0.9 | 0.9 |
|  | male | 58 | 52.7 | 100.0 | 14 | 45.2 | 100.0 |  |  |  |  |
| p = 0.543 | all | 110 | 100.0 |  | 31 | 100.0 |  |  |  |  |  |
| Age | <45 | 110 | 100.0 | 100.0 | 0 | 0.0 | 0.0 |  |  |  |  |
|  | ≥45 | 0 | 0.0 | 100.0 | 31 | 100.0 | 100.0 |  |  |  |  |
| p < 0.0001 | all | 110 | 100.0 |  | 31 | 100.0 |  |  |  |  |  |
| Histology | EN | 80 | 72.7 | 72.7 | 3 | 9.7 | 9.7 | 0.6 | 0.2 | 0.1 | 0.7 |
|  | other | 30 | 27.3 | 100.0 | 28 | 90.3 | 100.0 |  |  |  |  |
| p < 0.0001 | all | 110 | 100.0 |  | 31 | 100.0 |  |  |  |  |  |
| B symptoms | No | 66 | 60.5 | 60.5 | 15 | 48.4 | 48.4 | 0.4 | 0.5 | 0.007 | 0.01 |
|  | Yes | 43 | 39.5 | 100.0 | 16 | 51.6 | 100.0 |  |  |  |  |
| p = 0.302 | all | 109 | 100.0 |  | 31 | 100.0 |  |  |  |  |  |
| Bulky mass | No | 82 | 74.5 | 74.5 | 30 | 96.8 | 96.8 | 0.4 | -- | 0.8 | 0.4 |
|  | Yes | 28 | 25.4 | 100.0 | 1 | 3.2 | 100.0 |  |  |  |  |
| p = 0.0051 | all | 110 | 100.0 |  | 31 | 100.0 |  |  |  |  |  |
| Anemia, Hb levels less than 10^5^ g/L | No | 89 | 80.9 | 80.9 | 21 | 67.7 | 67.7 | 0.3 | 0.028 | 0.002 | 0.02 |
|  | Yes | 21 | 19.1 | 100.0 | 10 | 32.3 | 100.0 |  |  |  |  |
| p = 0.142 | all | 110 | 100.0 |  | 31 | 100.0 |  |  |  |  |  |
| Leukocytosis, oret han 15X10^9^/L | No | 94 | 86.2 | 86.2 | 29 | 93.5 | 93.5 | 0.8 | -- | 0.2 | 0.06 |
|  | Yes | 15 | 13.8 | 100.0 | 2 | 6.5 | 100.0 |  |  |  |  |
| p = 0.362 | all | 109 | 100.0 |  | 31 | 100.0 |  |  |  |  |  |
| Lymphocytopenia, <0.6 X 10^9^/L or <8% of WBC | No | 97 | 89.8 | 89.8 | 24 | 80.0 | 80.0 | 0.8 | 0.008 | 0.2 | 0.03 |
|  | Yes | 11 | 10.2 | 100.0 | 6 | 20.0 | 100.0 |  |  |  |  |
| p = 0.205 | all | 108 | 100.0 |  | 30 | 100.0 |  |  |  |  |  |
| Hypoalbuminemia, <40 g/L | No | 71 | 69.6 | 69.6 | 15 | 50.0 | 50.0 | 0.4 | 0.2 | 0.1 | 0.004 |
|  | Yes | 31 | 30.4 | 100.0 | 15 | 50.0 | 100.0 |  |  |  |  |
| p = 0.053 | all | 102 | 100.0 |  | 30 | 100.0 |  |  |  |  |  |
| High LDH level, >450 UI/L | No | 82 | 75.2 | 75.2 | 17 | 56.7 | 56.7 | 0.4 | 0.4 | 0.8 | 0.1 |
|  | Yes | 27 | 24.8 | 100.0 | 13 | 43.3 | 100.0 |  |  |  |  |
| p = 0.0672 | all | 109 | 100.0 |  | 30 | 100.0 |  |  |  |  |  |
| High B-2-microglobulin level, >25 mg/L | No | 73 | 85.9 | 85.9 | 11 | 42.3 | 42.3 | 0.5 | 0.06 | 0.3 | 0.09 |
|  | Yes | 12 | 14.1 | 100.0 | 15 | 57.7 | 100.0 |  |  |  |  |
| p < 0.0001 | all | 85 | 100.0 |  | 26 | 100.0 |  |  |  |  |  |
| Stage | Early(I‐II) | 75 | 68.8 | 68.8 | 11 | 35.5 | 35.5 | 0.2 | 0.7 | 0.03 | 0.02 |
|  | Advanced (III‐IV) | 34 | 31.2 | 100.0 | 20 | 64.5 | 100.0 |  |  |  |  |
| p = 0.0014 | all | 109 | 100.0 |  | 31 | 100.0 |  |  |  |  |  |
| Treatment | ABVD | 57 | 52.3 | 52.3 | 16 | 55.2 | 55.2 | 0.6 | 0.6 | 0.7 | 0.01 |
|  | MOPABV | 46 | 42.2 | 94.5 | 7 | 24.1 | 79.3 |  |  |  |  |
|  | MOPP | 5 | 4.6 | 99.1 | 3 | 10.3 | 89.7 |  |  |  |  |
|  | Other | 1 | 0.9 | 100.0 | 3 | 10.3 | 100.0 |  |  |  |  |
| p = 0.024 | all | 109 | 100.0 |  | 29 | 100.0 |  |  |  |  |  |
| EBV | No | 53 | 68.0 | 68.0 | 12 | 44.4 | 44.4 | 0.5 | 0.7 | 0.3 | 0.7 |
|  | Yes | 25 | 32.0 | 100.0 | 15 | 55.6 | 100.0 |  |  |  |  |
| p = 0.039 | all | 78 | 100.0 |  | 27 | 100.0 |  |  |  |  |  |
| Neutropenia toxicity | No | 77 | 70.0 | 70.0 | 17 | 54.8 | 54.8 | 0.7 | 0.3 | 0.02 | 0.4 |
|  | Yes | 33 | 30.0 | 100.0 | 14 | 45.2 | 100.0 |  |  |  |  |
| p = 0.133 | all | 110 | 100.0 |  | 31 | 100.0 |  |  |  |  |  |
| Anemia toxicity | No | 110 | 100.0 | 100.0 | 25 | 80.7 | 80.7 | --- | 0.9 | --- | 0.1 |
|  | Yes | 0 | 0.0 | 100.0 | 6 | 19.4 | 100.0 |  |  |  |  |
| p < 0.0001 | all | 110 | 100.0 |  | 31 | 100.0 |  |  |  |  |  |
| Thrombocytopenia toxicity | No | 108 | 98.2 | 98.2 | 27 | 87.1 | 87.1 | 0.6 | 0.9 | 0.7 | 0.1 |
|  | Yes | 2 | 1.8 | 100.0 | 4 | 12.9 | 100.0 |  |  |  |  |
| p = 0.021 | all | 110 | 100.0 |  | 31 | 100.0 |  |  |  |  |  |
| Pulmonary toxicity | No | 105 | 95.5 | 95.5 | 28 | 93.3 | 93.3 | 0.9 | 0.03 | 0.6 | 0.8 |
|  | Yes | 5 | 4.5 | 100.0 | 2 | 6.7 | 100.0 |  |  |  |  |
| p = 0.642 | all | 110 | 100.0 |  | 30 | 100.0 |  |  |  |  |  |
| Neurological toxicity | No | 90 | 81.8 | 81.8 | 28 | 90.3 | 90.3 | 0.9 | 0.5 | 0.9 | 0.08 |
|  | Yes | 20 | 18.2 | 100.0 | 3 | 9.7 | 100.0 |  |  |  |  |
| p = 0.408 | all | 110 | 100.0 |  | 31 | 100.0 |  |  |  |  |  |
| Infectious toxicity | No | 73 | 66.4 | 66.4 | 17 | 54.8 | 54.8 | 0.7 | 0.1 | 0.1 | 0.1 |
|  | Yes | 37 | 33.6 | 100.0 | 14 | 45.2 | 100.0 |  |  |  |  |
| p = 0.291 | all | 110 | 100.0 |  | 31 | 100.0 |  |  |  |  |  |
